# Cardiotoxicity of newer HER2-Directed therapies in breast cancer: A systematic review and meta-analysis of incidence and comparative risk

**DOI:** 10.1016/j.breast.2026.104880

**Published:** 2026-07-20

**Authors:** Senthamizhan Sundaramoorthy, Narendhar Gokulanathan, Rona Joseph P, Anoop Tm, Sherin P. Mathew, Harpreet Kaur, Thamizharuvi Muthukumarasamy, Joe Jose, Kalai Bharathi Jayaraman, Parvathy Rajmohan, Vyshak Thovarayi, Aswin Sathyadas, Shibu V. Ignatious, Sariga Ajit Valaparambil

**Affiliations:** a Department of Medical Oncology, Regional Cancer Centre, Trivandrum, Kerala, India; b Department of Medical Oncology, Apollo Hospitals, Bangalore, India; c Department of Public Health, Postgraduate Institute of Medical Education and Research (PGIMER), Chandigarh, India; d Department of Community Medicine, All India Institute of Medical Sciences (AIIMS), New Delhi, India

**Keywords:** HER2-Targeted therapy, Antibody–drug conjugates, Cardiotoxicity, Breast cancer, Left ventricular ejection fraction

## Abstract

**Purpose:**

This systematic review and meta-analysis aimed to estimate the incidence and comparative risk of cardiotoxicity associated with newer HER2-targeted therapies compared with conventional trastuzumab-based regimens. It also evaluates the differences across drug classes, clinical subgroups, and monitoring strategies.

**Materials and methods:**

PubMed, Embase, CENTRAL, and Scopus were searched (2005-2025) for randomized controlled trials (RCTs) and prospective studies reporting cardiac outcomes with HER2-directed therapies. Pooled cardiotoxicity incidence was calculated from single-arm studies and intervention arms of RCTs and risk ratios (RRs) from RCTs using random-effects models. Subgroup analyses were performed by drug class, anthracycline exposure, HER2 category, age, and cardiac monitoring frequency. Certainty of evidence was assessed using GRADE.

**Results:**

Fifty-eight studies (35,984 patients) were included. Pooled cardiotoxicity incidence was 5.08% (95% CI, 3.37 to 7.58) with left ventricular ejection fraction (LVEF) decline in 4.68% patients, and other cardiac events were each reported in <1% patients. Across 32 RCTs, no significant difference in cardiotoxicity was observed between newer HER2-directed therapies and trastuzumab-based comparator regimens (including trastuzumab monotherapy or trastuzumab combined with chemotherapy, with or without pertuzumab) (RR, 0.97; 95% CI, 0.73 to 1.29). Subgroup analyses showed variability across study characteristics, with higher incidence observed in studies using more frequent cardiac monitoring (8.15%; 95% CI, 3.13 to 19.61%, P > 0.05).

**Conclusion:**

Newer HER2-directed therapies demonstrate low cardiotoxicity, with rare serious events and no evidence of excess risk compared with conventional trastuzumab-based regimens. These findings support cardiac safety but suggest the need for standardized monitoring and uniform cardiotoxicity definitions.

## Highlights

• Cardiotoxicity incidence with HER2-directed therapies is low (∼5%) across studies.
• Serious cardiac events are rare, with most outcomes occurring in <1% of patients.
• No increased cardiotoxicity risk compared with trastuzumab-based regimens.
• Cardiotoxicity estimates vary by drug class, treatment strategy, and monitoring frequency.
• More frequent cardiac monitoring is associated with higher reported event rates.

## Graphical abstract

**Figure:**
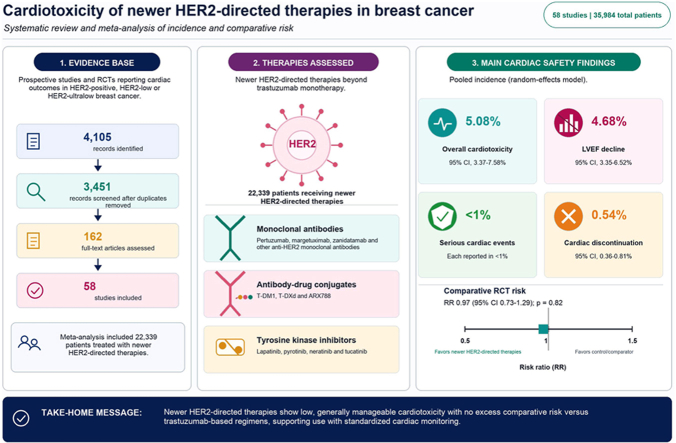

## 1 Introduction

Breast cancer is the most commonly diagnosed malignancy among women and a leading cause of cancer death worldwide, with approximately 670,000 deaths in 2022 [1]. Approximately 15-20% of breast cancers overexpress the human epidermal growth factor receptor 2 (HER2), which is a molecular subtype associated with aggressive behaviour and poor prognosis of breast cancer [2]. With the introduction of HER2-directed therapy, the treatment landscape has been transformed, which turned HER2-positive breast cancer into a far more treatable disease with markedly improved survival outcomes [3].

The approval of trastuzumab, a monoclonal antibody directed against the HER2 receptor, in 1998 represented a milestone in the treatment of HER2-positive breast cancer [4]. However, primary and acquired resistance to trastuzumab led to the development of additional HER2-directed therapies aimed to enhance clinical outcomes. These include newer monoclonal antibodies (e.g., pertuzumab), tyrosine kinase inhibitors (TKIs), and antibody–drug conjugates (ADCs). These agents represent therapeutic strategies beyond trastuzumab monotherapy and are now widely incorporated into standard clinical practice [5,6].

Despite these advances, cardiotoxicity remains an important limitation of HER2-directed therapies. The HER2 signalling pathway plays a crucial role in preserving normal heart function and its blockage can lead to left ventricular systolic dysfunction (LVSD) or even clinical heart failure. A decline in left ventricular ejection fraction (LVEF) may lead to treatment interruption or discontinuation and therefore prevents patients from receiving the full benefit of therapy [[7], [8], [9]]. Research suggests that these newer HER2-directed therapies, including pertuzumab and ADCs such as trastuzumab emtansine (T-DM1) and trastuzumab deruxtecan (T-DXd), may be associated with a lower incidence of cardiotoxicity. For instance, pooled analyses reported symptomatic cardiac events in less than 2% of patients treated with these agents [10,11]. However, differences in study design, length of follow-up, and cardiotoxicity definitions make it challenging to directly compare risks across drug classes.

The expanding use of these newer HER2-directed therapies has broadened the treatment options and improved the clinical outcomes, but their long-term cardiac effects are not fully understood. Most of the available data are derived from heterogeneous trials that differ in sample size, cardiac monitoring frequency, and cardiotoxicity definitions. Therefore, this systematic review and meta-analysis aim to summarise the incidence and comparative risk of cardiotoxicity associated with newer HER2-directed therapies compared with conventional trastuzumab-based comparator regimens (including trastuzumab monotherapy or trastuzumab combined with chemotherapy, with or without pertuzumab). These findings will help refine treatment decisions, guide cardiac monitoring practices, and highlight areas where further research is needed.

## 2 Materials and Methods

This systematic review was conducted in accordance with the Preferred Reporting Items for Systematic Reviews and Meta-Analyses (PRISMA) 2020 statement [12]. The protocol was registered in the International Prospective Register of Systematic Reviews (PROSPERO): CRD420251146525.

### 2.1 Inclusion criteria

We included clinical studies evaluating the cardiac safety of newer HER2-directed therapies in adult patients (≥18 years) with a histologically or cytologically confirmed diagnosis of breast cancer classified as HER2-positive, HER2-low, or HER2-ultralow. The eligible study designs were randomized controlled trials (RCTs; phase II–IV), prospective cohort studies, and single-arm clinical trials administering at least one HER2-directed agent, including ADCs, monoclonal antibodies, or TKIs.

The included studies were required to report at least one cardiac safety outcome, such as symptomatic heart failure, LVEF decline, QTc prolongation, arrhythmias, myocarditis, cardiac death, major adverse cardiac events (MACE), or treatment modification (dose reduction, interruption, or discontinuation) attributed to cardiac toxicity. This review focused on HER2-directed therapies beyond trastuzumab monotherapy. Studies evaluating trastuzumab monotherapy alone were not included unless used as a comparator. For comparative analyses, control groups included conventional trastuzumab-based regimens (trastuzumab monotherapy or trastuzumab combined with chemotherapy, with or without pertuzumab).

The primary objective was to estimate the pooled incidence and relative risk of cardiotoxicity associated with newer HER2-directed therapies in breast cancer. The secondary objective was to explore potential effect modifiers and clinical variables such as age, prior anthracycline exposure, HER2 disease category, type of therapy used, patient age, and cardiac monitoring frequency.

### 2.2 Definitions of cardiotoxicity symptoms

Symptomatic heart failure was defined as a clinical diagnosis of heart failure based on Common Terminology Criteria for Adverse Events (CTCAE) grading or study-specific criteria. LVEF decline was defined as a protocol-specified decrease, such as a ≥10% reduction from baseline to below 50%, or the study-defined threshold. QTc prolongation was defined as QTc ≥480 ms, ≥grade 3 per CTCAE, or according to study criteria. Arrhythmias included any newly diagnosed atrial or ventricular arrhythmia or conduction abnormality. Myocarditis was defined by clinical diagnosis, imaging, or biopsy confirmation, based on each study's defined criteria. Cardiac death was defined as mortality directly attributed to a cardiac cause. MACE was defined as a composite of cardiovascular death, non-fatal myocardial infarction, non-fatal stroke, or hospitalization for heart failure, when reported. Treatment discontinuation or interruption due to cardiac toxicity was defined as any dose reduction, temporary treatment hold, or permanent discontinuation directly attributed to a cardiac adverse event.

### 2.3 Search strategy

PubMed, Embase, the Cochrane Central Register of Controlled Trials (CENTRAL), and Scopus were searched to identify eligible studies. A comprehensive literature search was conducted using free-text terms and Medical Subject Headings (MeSH), covering the period from January 2005 to September 2025 and restricted to articles published in English. The search strategy included terms related to HER2 breast cancer; HER2-directed therapies such as ADCs, monoclonal antibodies, and TKIs; and cardiac safety outcomes. The complete search strategy for each database is provided in the supplementary file.

### 2.4 Data collection and analysis

Two reviewers independently screened the articles collected from titles and abstracts of the studies. Full-text versions of potentially eligible studies were independently assessed for final inclusion by two reviewers, and any disagreements were resolved by discussion with a third reviewer. Two review authors independently extracted the following data: study identifiers, year of publication, study design, number and characteristics of patients, type of HER2-directed therapy, treatment duration, follow-up period, cardiac monitoring method, and definitions and outcomes of cardiotoxicity in each study.

Two authors independently evaluated the risk of bias. RCTs were assessed using the Cochrane Risk of Bias 2 (RoB 2) tool [13], and non-randomized studies were assessed using the ROBINS-I tool [14]. Each domain was rated as having low risk, moderate risk, or high risk of bias, and disagreements were resolved by consensus. Risk-of-bias summaries were generated using the robvis tool [15].

We collected the quantitative data for meta-analysis where appropriate. The pooled incidence of cardiotoxicity (from single-arm studies and intervention arms of RCTs) was estimated using a random-effects model (DerSimonian–Laird) and expressed per 100 patients with 95% confidence intervals (CIs). We also calculated pooled incidence for each individual cardiotoxicity outcome using random-effects models. As the definitions of cardiotoxicity outcomes varied across studies, including the differences in thresholds for LVEF decline, QTc prolongation criteria, myocarditis definitions, arrhythmia classification, and criteria for treatment discontinuation, we extracted each definition as reported in the study and harmonized them where feasible.

We performed comparative analyses by pooling risk ratios (RRs) with 95% CIs from RCTs comparing newer HER2-directed therapies with conventional trastuzumab-based comparator regimens using random-effects meta-analysis. When hazard ratios (HRs) were reported, these were pooled separately. Statistical heterogeneity was assessed using the I^2^ and τ^2^ statistics, and prediction intervals were reported where appropriate.

We performed the sensitivity analyses by excluding studies at high risk of bias, abstract-only studies, and studies with short follow-up (<3 months). We decided to visually inspect the funnel plots to indicate publication bias and statistically use Egger's regression test when at least ten studies were available for an outcome. All statistical analyses were conducted in R (meta, metafor, and dmetar packages) [16].

### 2.5 Subgroup analysis

We conducted the subgroup analyses based on pre-specified clinical and treatment-related variables. Subgroups were analysed according to drug class (ADCs, TKIs, or monoclonal antibodies), individual therapeutic agent, monotherapy versus combination therapy, prior anthracycline exposure, HER2 expression category (positive vs low), patient age group (<55 or ≥55 years), and frequency of cardiac monitoring (≤8–9 or >9 weeks). For each subgroup, pooled estimates were calculated using random-effects models.

### 2.6 Grading of evidence

We assessed the overall certainty of the evidence for cardiotoxicity outcomes using the five GRADE domains, such as risk of bias, inconsistency, indirectness, imprecision, and publication bias, according to the GRADE approach. The certainty of evidence for each outcome was categorized as high, moderate, low, or very low [17]. The findings were summarized in a Summary of Evidence table [18].

## 3 Results

The database searches initially identified 4105 records: 1741 from PubMed, 1454 from Embase, 305 from Scopus, and 605 from Cochrane CENTRAL. After removing 654 duplicates, 3451 studies remained for title and abstract screening. Of these, 3289 records were excluded due to retrospective design, case reports or case series with fewer than 10 patients, conference abstracts without extractable data, or editorial/commentary formats. A total of 162 full-text articles were assessed for eligibility.

**Fig. 1:**
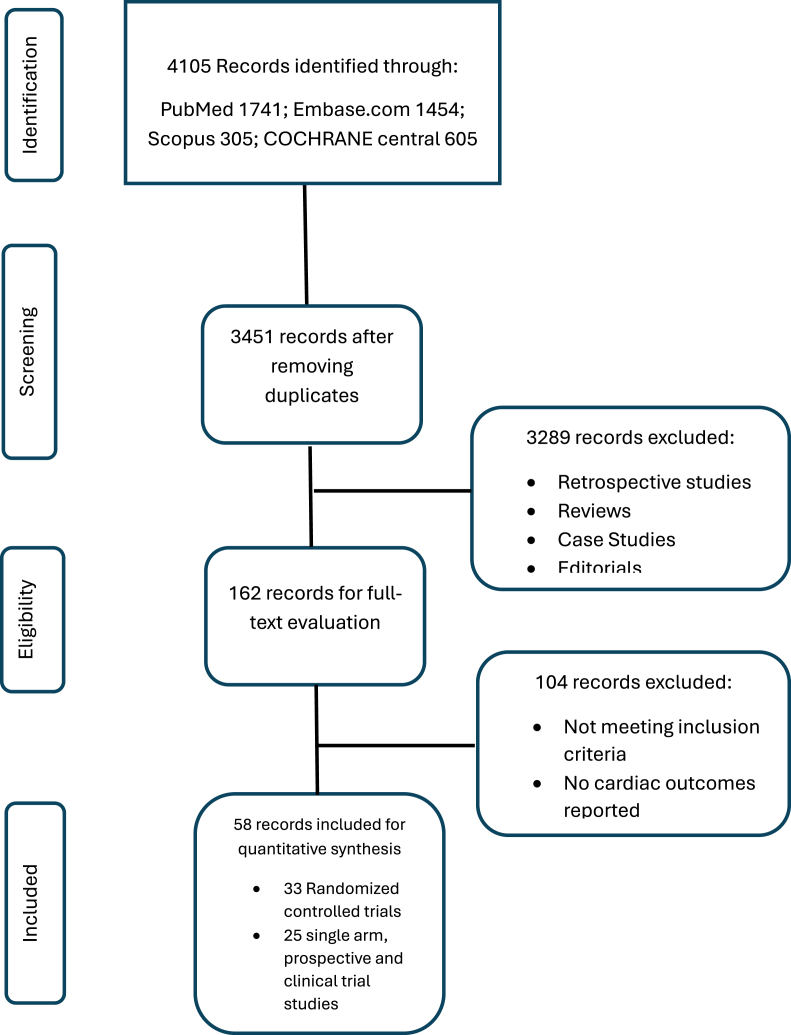
PRISMA flow diagram of study selection.

Of these, 104 studies were excluded because they did not meet the inclusion criteria or did not report any cardiac safety outcomes. Ultimately, 58 studies met the eligibility criteria and were included in the quantitative synthesis (meta-analysis) (Fig. 1). These consisted of 33 RCTs [[19], [20], [21], [22], [23], [24], [25], [26], [27], [28], [29], [30], [31], [32], [33], [34], [35], [36], [37], [38], [39], [40], [41], [42], [43], [44], [45], [46], [47], [48], [49], [50], [51]] and 25 single-arm prospective clinical trial studies [11,[52], [53], [54], [55], [56], [57], [58], [59], [60], [61], [62], [63], [64], [65], [66], [67], [68], [69], [70], [71], [72], [73], [74], [75]].

Across the included studies, a total of 35,984 patients contributed to the pooled analyses. This included 14,410 patients in the experimental arms and 13,645 patients in the comparator arms of the RCTs, along with 7929 patients from single-arm studies. A total of 2319 cardiotoxicity events were reported, including 1631 in RCTs and 688 in single-arm studies. Median age of patients ranged from 46 to 76 years and median follow-up duration from 5.2 to 117.6 months across the studies.

The HER2-directed therapies evaluated include monoclonal antibodies (trastuzumab-containing regimens and other agents such as pertuzumab, margetuximab, and zanidatamab), ADCs (T-DM1, T-DXd, and ARX788), and TKIs (lapatinib, pyrotinib, neratinib, and tucatinib). Among patients receiving newer HER2-directed therapies (n = 22,339), 7772 received monoclonal antibodies, 5864 received ADCs, 8450 received TKIs, and 253 received combination regimens. In RCTs, 4553 patients received monoclonal antibodies, 5002 received ADCs, 4632 received TKIs, and 223 received combination regimens, while in single-arm studies, 3219; 862; 3818 and 30 patients received these therapies, respectively. Most of the studies included HER2-positive populations (n = 56) [[19], [20], [21], [22], [23], [24], [25],[52], [53], [54], [55], [56], [57], [58], [59], [60], [61], [62], [63], [64][19–25,52–64,[40], [41], [42], [43], [44], [45], [46], [47], [48], [49], [50], [51], [52], [53], [54], [55], [56], [57], [58], [59], [60], [61], [62], [63], [64], [65], [66], [67], [68], [69],[71], [72], [73], [74], [75]], while a smaller number enrolled HER2-low patients (n = 2) [26,47]. Cardiac monitoring frequency varied across the studies, with longer intervals (>9 weeks) as the most reported. The detailed characteristics of the included studies are summarized in Table 1.

**Table 1:** Study characteristics.

Author	Year	Study	Study design	Drug Class	Median Follow-up (months)	Cardiac Outcomes	Frequency of cardiac monitoring	No. of patients, N (Exp)	No. of patients, N (Comp)	Cardiotoxicity events Exp n/N (%)	Cardiotoxicity events Comp n/N (%)
Hans Wildiers [19]	2022	EORTC 75111-10114	RCT	Monoclonal antibody	54	LVEF decline, Symptomatic Heart Failure; Cardiac death; Composite cardiac endpoint	≤8–9 weeks	41	39	8/41 (19.5%)	3/39 (7.7%)
Hope S. Rugo [20]	2022	SOPHIA	RCT	Monoclonal antibody	20.2	LVEF decline; Cardiac death	≤8–9 weeks	264	266	15/264 (5.7%)	16/266 (6.0%)
Binghe Xu [21]	2020	PUFFIN	RCT	Monoclonal antibody	13.7	LVEF decline; Symptomatic heart failure; Cardiac Death	>9 weeks	122	121	4/122 (3.3%)	0/121 (0.0%)
E. de Azambuja [23]	2023	APHINITY	RCT	Monoclonal antibody	74	LVEF decline; Cardiac Death; Symptomatic heart failure; Treatment discontinuation; Composite cardiac endpoint	>9 weeks	2364	2405	263/2364 (11.1%)	245/2405 (10.2%)
Anna van der Voort [24]	2021	TRAIN2	RCT	Monoclonal antibody	48.8	LVEF decline	≤8–9 weeks	221	217	133/221 (60.2%)	72/217 (33.2%)
Sandra M Swain [27]	2020	CLEOPATRA	RCT	Monoclonal antibody	99·9	LVEF decline; Symptomatic Heart Failure	≤8–9 weeks	408	396	2/408 (0.5%)	3/396 (0.8%)
Liang Huang [28]	2024	PEONY	RCT	Monoclonal antibody	62.9	LVEF decline; Composite cardiac endpoint	≤8–9 weeks	218	110	51/218 (23.4%)	23/110 (20.9%)
Amit Garg [29]	2013	Exposure–response analysis of pertuzumab in HER2-positive metastatic breast cancer: absence of effect on QTc prolongation and other ECG parameters	RCT	Monoclonal antibody	NR	QTc Prolongation	NR	20	17	0/20 (0.0%)	6/17 (35.3%)
Luca Gianni [37]	2011	NEOSPHERE	RCT	Monoclonal antibody	NR	LVEF decline; Symptomatic Heart Failure	NR	Arm A: 107	Arm B: 107 Arm C:108 Arm D: 94	Arm A: 3/107 (2.8%)	Arm B: 1/107 (0.9%) Arm C: 2/108 (1.9%) Arm D: 1/94 (1.1%)
A. Schneeweiss [38]	2013	TRYPHAENA	RCT	Monoclonal antibody	20	LVEF decline	>9 weeks	Arm A: 68	Arm B: 65 Arm C: 67	Arm A: 4/68 (5.9%)	Arm B: 13/65 (20.0%) Arm C: 6/67 (9.0%)
Ander Urruticoechea [40]	2017	PHEREXA	RCT	Monoclonal antibody	28.6	LVEF decline; Cardiac Death	NR	228	218	20/228 (8.8%)	9/218 (4.1%)
Joyce O'Shaughnessy [41]	2021	PHRANCESCa	RCT	Monoclonal antibody	NR	LVEF decline; Arrythmia; Symptomatic Heart Failure; Treatment discontinuation	>9 weeks	160	NR	10/160 (6.2%)	NR
Antoinette R Tan [42]	2020	FEDERICA	RCT	Monoclonal antibody	Ongoing	LVEF decline; Symptomatic Heart Failure	≤8–9 weeks	252	248	9/252 (3.6%)	7/248 (2.8%)
Xia W [48]	2024	METIS	RCT	Monoclonal antibody	16.3	LVEF decline	NR	80	76	0/80 (0.0%)	1/76 (1.3%)
Sara A Hurvitz [39]	2017	KRISTINE	RCT	Monoclonal antibody + ADC	36	LVEF decline; Symptomatic Heart Failure; Cardiac Death	≤8–9 weeks	223	219	0/223 (0.0%)	1/219 (0.5%)
Fabrice André [32]	2023	DESTINY-BREAST02	RCT	ADC	21·5	LVEF decline; Symptomatic Heart Failure; Treatment discontinuation; Composite cardiac endpoint	≤8–9 weeks	371	184	22/371 (5.9%)	5/184 (2.7%)
Javier Cortés [33]	2024	DESTINY-BREAST03	RCT	ADC	41	LVEF decline	>9 weeks	257	261	11/257 (4.3%)	4/261 (1.5%)
Sunil Verma [34]	2012	EMILIA	RCT	ADC	24·1	LVEF decline	>9 weeks	481	445	12/481 (2.5%)	10/445 (2.2%)
Romualdo Barroso-Sousa [35]	2022	ATEMPT	RCT	ADC	46.8	LVEF decline	>9 weeks	383	114	7/383 (1.8%)	7/114 (6.1%)
Ian E. Krop [36]	2021	KAITLIN	RCT	ADC	57.1	Composite cardiac endpoint	>9 weeks	912	926	28/912 (3.1%)	63/926 (6.8%)
Xichun Hu [45]	2025	ACE-BREAST-02	RCT	ADC	NR	Arrythmia	≤8–9 weeks	220	215	0/220 (0.0%)	1/215 (0.5%)
Sara A Hurvitz [46]	2013	HURVITZ	RCT	ADC	23	LVEF decline; Symptomatic Heart Failure	>9 weeks	69	66	3/69 (4.3%)	3/66 (4.5%)
A. Bardia [47]	2024	DESTINY-BREAST06	RCT	ADC	18.2	LVEF decline; Symptomatic Heart Failure	>9 weeks	434	417	35/434 (8.1%)	19/417 (4.6%)
Ian E Krop [50]	2014	TH3RESA	RCT	ADC	7·2	LVEF decline	>9 weeks	403	184	6/403 (1.5%)	2/184 (1.1%)
Edith A. Perez [51]	2017	MARIANNE	RCT	ADC	35	LVEF decline	≤8–9 weeks	Arm A: 361	Arm B: 353 Arm C: 366	Arm A: 3/361 (0.8%)	Arm B: 16/353 (4.5%) Arm C: 9/366 (2.5%)
S. Modi [26]	2022	DESTINY-BREAST04	RCT	ADC	18.4	LVEF decline, Symptomatic Heart Failure	≤8–9 weeks	371	172	73/371 (19.7%)	10/172 (5.8%)
G. von Minckwitz [25]	2019	KATHERINE	RCT	ADC	41.4	LVEF decline; Treatment discontinuation; Composite cardiac endpoint	>9 weeks	740	720	10/740 (1.4%)	14/720 (1.9%)
Binghe Xu [43]	2021	PHOEBE	RCT	TKI	21·8	QTc prolongation; Arrythmia; Composite cardiac endpoint	>9 weeks	134	132	10/134 (7.5%)	4/132 (3.0%)
Miguel Martin [44]	2017	EXTENET	RCT	TKI	5·2	MACE; Cardiac death	NR	1408	1408	7/1408 (0.5%)	1/1408 (0.1%)
Cristina Saura [30]	2020	NALA	RCT	TKI	29.9	Arrythmias, LVEF decline, QTc prolongation, MACE	>9 weeks	303	311	32/303 (10.6%)	32/311 (10.3%)
R.K. Murthy [31]	2020	HER2CLIMB	RCT	TKI	14	Cardiac death	>9 weeks	404	197	2/404 (0.5%)	2/197 (1.0%)
E. de Azambuja [22]	2024	ALTTO	RCT	TKI	117.6	Cardiac Death; Composite cardiac endpoint	>9 weeks	2061	2076	105/2061 (5.1%)	122/2076 (5.9%)
Karen A. Gelmon [49]	2015	MA.31	RCT	TKI	21.5	LVEF decline; Symptomatic Heart Failure; Cardiac Death; Arrythmia	>9 weeks	322	325	1/322 (0.3%)	9/325 (2.8%)
Ji-Xin Yang [52]	2024	BRECAN	Single arm	Monoclonal antibody	11.2	LVEF decline; Symptomatic Heart Failure; Cardiac Death	>9 weeks	96	NA	8/96 (8.3%)	NA
Chia C. Portera [53]	2008	PORTERA	Single arm	Monoclonal antibody	NR	LVEF decline; Symptomatic Heart Failure; Cardiac Death	≤8–9 weeks	11	NA	6/11 (54.5%)	NA
Toshinari Yamashita [54]	2021	JBCRG-M03	Single arm	Monoclonal antibody	NR	LVEF decline	>9 weeks	49	NA	0/49 (0.0%)	NA
Joaquín Gavilá [55]	2019	Opti-HER HEART	Single arm	Monoclonal antibody	NR	LVEF decline	>9 weeks	83	NA	2/83 (2.4%)	NA
Chau Dang [58]	2022	BERENICE	Prospective Cohort	Monoclonal antibody	64.1	LVEF decline; Symptomatic Heart Failure; Treatment discontinuation	>9 weeks	397	NA	87/397 (21.9%)	NA
Mirela Gherghe [59]	2022	Evaluating Cardiotoxicity in Breast Cancer Patients Treated with HER2 Inhibitors: Could a Combination of Radionuclide Ventriculography and Cardiac Biomarkers Predict the Cardiac Impact?	Prospective Cohort	Monoclonal antibody	NR	LVEF decline	NR	22		5/22 (22.7%)	NA
Chunyu Tian [60]	2024	GDF-15 is a potential candidate biomarker for an elevated risk of cardiotoxicity in breast cancer patients receiving neoadjuvant dual anti-HER2 therapy	Prospective Cohort	Monoclonal antibody	NR	LVEF decline	NR	103	NA	7/103 (6.8%)	NA
Anthony F. Yu [62]	2023	Association of Circulating Cardiomyocyte Cell-Free DNA With Cancer Therapy–Related Cardiac Dysfunction in Patients Undergoing Treatment for ERBB2-Positive Breast Cancer	Prospective Cohort	Monoclonal antibody	12	LVEF decline; Symptomatic Heart Failure; Cardiac Death	NR	71	NA	10/71 (14.1%)	NA
D. Lenihan [64]	2011	Pooled analysis of cardiac safety in patients with cancer treated with pertuzumab	Single arm	Monoclonal antibody	NR	LVEF decline; Symptomatic Heart Failure	NR	331	NA	39/331 (11.8%)	NA
Santiago Escrivá-de-Romani [69]	2025	Zanidatamab plus palbociclib and fulvestrant in previously treated patients with hormone receptor-positive, HER2-positive metastatic breast cancer: primary results from a two-part, multicentre, single-arm, phase 2a study	Single arm	Monoclonal antibody	16·1	LVEF decline	NR	51	NA	10/51 (19.6%)	NA
Edith A. Perez [70]	2016	VELVET	Single arm	Monoclonal antibody	27.8	LVEF decline; Symptomatic Heart Failure; Cardiac death; Arrythmia; Composite cardiac endpoint	NR	106	NA	20/106 (18.9%)	NA
Sherko Kuemmel [71]	2021	MetaPHER	Single arm	Monoclonal antibody	27	LVEF decline; Symptomatic Heart Failure; Composite cardiac endpoint	NR	412	NA	44/412 (10.7%)	NA
Sainan Cheng [72]	2023	Longitudinal assessment of cardiac parameters through MRI in breast cancer patients treated with anti-HER2 therapy	Prospective cohort	Monoclonal antibody	NR	LVEF decline; Symptomatic Heart Failure; Composite cardiac endpoint	≤8–9 weeks	24	NA	7/24 (29.2%)	NA
D. Miles [74]	2021	PERUSE	Single arm	Monoclonal antibody	68.7	LVEF decline; Symptomatic Heart Failure; Treatment discontinuation	≤8–9 weeks	1436	NA	236/1436 (16.4%)	NA
V. Michalaki [75]	2017	MICHALAKI	Single arm	Monoclonal antibody	NR	LVEF decline	NR	27	NA	0/27 (0.0%)	NA
Katia Khoury [67]	2021	SAFE-HEaRt	Single arm	Monoclonal antibody + ADC	42	LVEF decline; Symptomatic Heart Failure	>9 weeks	30	NA	19/30 (63.3%)	NA
Rea Levicki [56]	2023	Effects of trastuzumab and trastuzumab emtansine on corrected QT interval and left ventricular ejection fraction in patients with metastatic (HER2+) breast cancer	Prospective cohort	ADC	6	LVEF decline; QTc prolongation	>9 weeks	26	NA	2/26 (7.7%)	NA
Ian E. Krop [63]	2015	Feasibility and Cardiac Safety of Trastuzumab Emtansine After Anthracycline-Based Chemotherapy As (neo)Adjuvant Therapy for Human Epidermal Growth Factor Receptor 2–Positive Early-Stage Breast Cancer	Single arm	ADC	24.6	LVEF decline; Symptomatic Heart Failure; Cardiac Death; Treatment discontinuation	>9 weeks	148	NA	5/148 (3.4%)	NA
S. Modi [11]	2019	DESTINY-BREAST01	Single arm	ADC	11.1	LVEF decline; Symptomatic Heart Failure; Treatment discontinuation	≤8–9 weeks	184	NA	3/184 (1.6%)	NA
Nadia Harbeck [73]	2024	DESTINY-BREAST12	Single arm	ADC	15.4	LVEF decline; Treatment discontinuation; Composite cardiac endpoint	>9 weeks	504	NA	64/504 (12.7%)	NA
Patrick G. Morris [57]	2013	Long-Term Cardiac Safety and Outcomes of Dose-Dense Doxorubicin and Cyclophosphamide Followed by Paclitaxel and Trastuzumab With and Without Lapatinib in Patients With Early Breast Cancer	Single arm	TKI	84.0	Symptomatic Heart Failure; Cardiac Death	NR	165	NA	5/165 (3.0%)	NA
Wei Wang [61]	2022	Cardiotoxicity monitoring of pyrotinib in combination with nab-paclitaxel, doxorubicin, and cyclophosphamide in HER2-positive breast cancer: a single-armed clinical trial	Single arm	TKI	NR	LVEF decline; Symptomatic Heart Failure; Cardiac Death; QTc prolongation	NR	22	NA	9/22 (40.9%)	NA
Roberto A. Leon-Ferre [65]	2020	Alliance	Single arm	TKI	63.6	LVEF decline; Symptomatic Heart Failure; Cardiac Death	>9 weeks	30	NA	4/30 (13.3%)	NA
Yuan Yuan [66]	2021	Phase II Study of Neratinib in Older Adults with HER2 Amplified or HER2/3 Mutated Metastatic Breast Cancer	Single arm	TKI	NR	LVEF decline	≤8–9 weeks	25	NA	1/25 (4.0%)	NA
Edith A. Perez [68]	2008	Cardiac Safety of Lapatinib: Pooled Analysis of 3689 Patients Enrolled in Clinical Trials	Pooled trials	TKI	NR	LVEF decline; Symptomatic Heart Failure; Composite cardiac endpoint	>9 weeks	3576	NA	95/3576 (2.7%)	NA

Cardiotoxicity (%) was calculated as the number of events divided by the total number of patients in each study arm. Definitions of cardiotoxicity varied across studies and are detailed in the ‘Cardiac Outcomes’ column.

ADC, antibody-drug conjugate; Comp, comparator; DNA, deoxyribonucleic acid; ECG, electrocardiograph; Exp, experimental arm; HER2, human epidermal growth factor receptor 2; LVEF, left ventricular ejection fraction; MACEs, major adverse cardiovascular events; MRI, magnetic resonance imaging; NA, not applicable; NR, not reported; QTc, corrected QT interval; RCT, randomized controlled trial; T-DXd, trastuzumab deruxtecan; TKI, tyrosine kinase inhibitor.

**Fig. 2:**
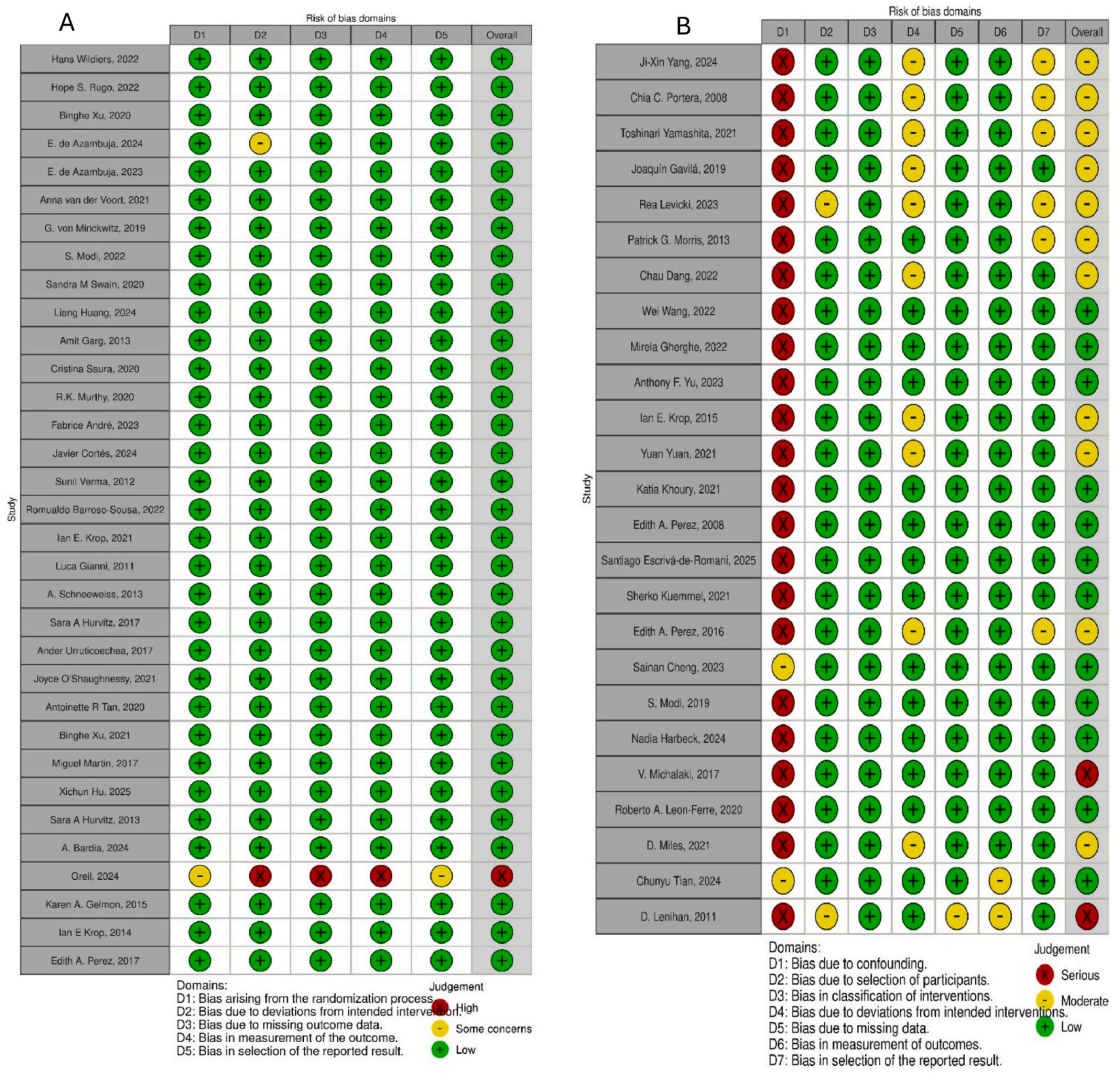
**Risk of bias assessment of included studies.** (A) Randomized controlled trials assessed using the Cochrane Risk of Bias 2 (RoB 2) tool and (B) non-randomized studies assessed using the Risk Of Bias In Non-randomized Studies of Interventions (ROBINS-I) tool. Each row represents an individual study, and columns represent bias domains with overall judgement. Green indicates low risk of bias, yellow indicates some concerns (RoB 2) or moderate risk (ROBINS-I), and red indicates high (RoB 2) or serious (ROBINS-I) risk of bias. (For interpretation of the references to colour in this figure legend, the reader is referred to the Web version of this article.)

Risk-of-bias assessment using the RoB 2 and ROBINS-I tools showed that 44 studies [11,[19], [20], [21], [22], [23], [24], [25], [26], [27], [28], [29], [30], [31], [32], [33], [34], [35], [36], [37], [38], [39], [40], [41], [42], [43], [44], [45], [46], [47],[49], [50], [51],[59], [60], [61], [62],[65], [66], [67], [68], [69], [70], [71], [72], [73], [74]] were at low risk of bias, 11 studies were at moderate risk [[52], [53], [54], [55], [56], [57], [58],^63^,^66^,^70^,^74^], and 3 studies were at high risk of bias [48,64,75] (Fig. 2a and b).

### 3.1 Effects of newer HER2-Directed therapies

#### 3.1.1 Overall cardiotoxicity

**Fig. 3:**
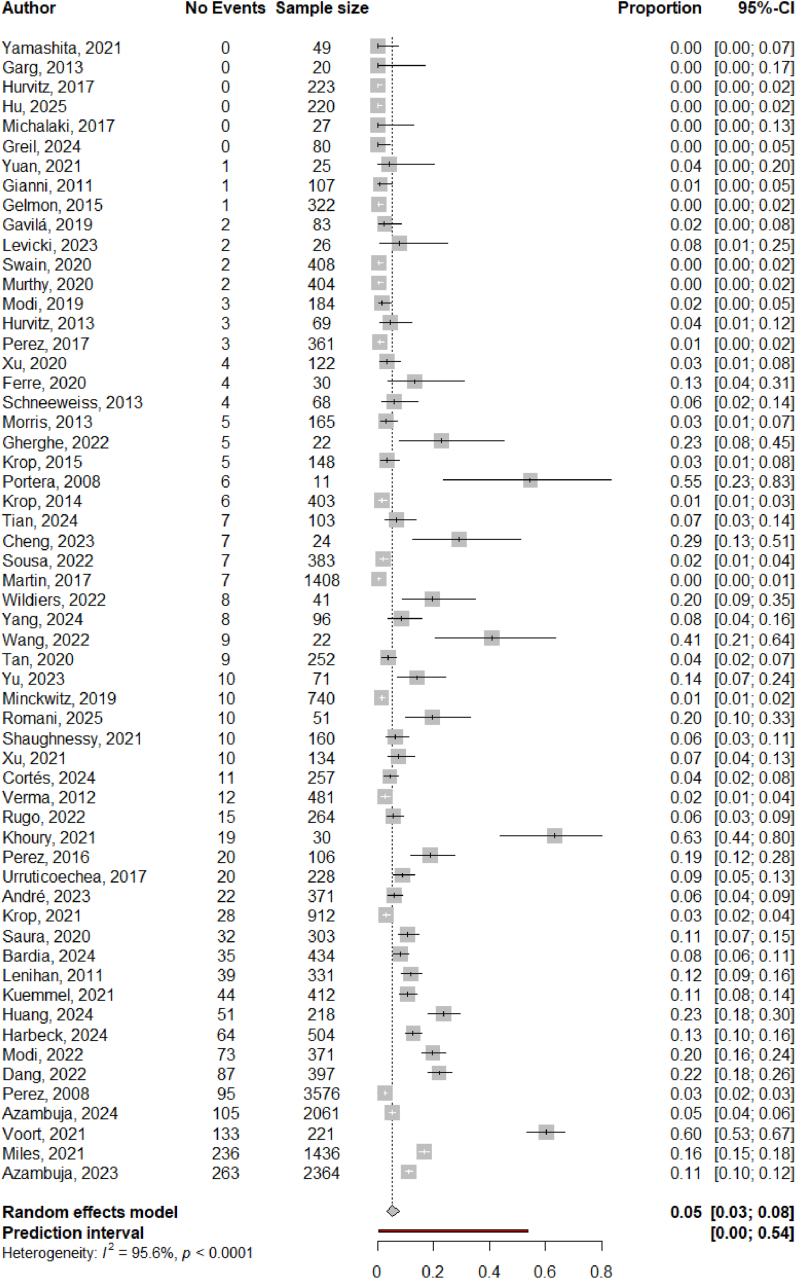
**Forest plot of pooled incidence of cardiotoxicity in patients receiving newer HER2-directed therapies.** Squares represent study-specific estimates with sizes proportional to inverse variance weighting, and horizontal lines indicate 95% confidence intervals. The diamond represents the pooled estimate using a random-effects model. The red line indicates the prediction interval. Heterogeneity was assessed using the I^2^ statistic. Cardiotoxicity definitions were based on individual study criteria. (For interpretation of the references to colour in this figure legend, the reader is referred to the Web version of this article.)

From pooling the data from 58 studies, including 22,339 patients who received newer HER2-directed therapies, we found a cardiotoxicity incidence of 5.08% (95% CI, 3.37% to 7.58%) with significant heterogeneity (I^2^ = 95.6%, τ^2^ = 2.3197) (Fig. 3). Detailed incidence of cardiotoxicity is summarized in Supplementary Table 1. Evidence of small-study effects was detected by Egger's test (p = 0.0258), consistent with asymmetry observed in the funnel plot **(**Supplementary Fig. 1**).**

#### 3.1.2 Individual cardiotoxicity outcomes

Pooled incidence estimates for specific cardiac outcomes showed that.

LVEF decline occurred in 4.68% of patients (95% CI, 3.35% to 6.52%). Events such as symptomatic heart failure, QTc prolongation, arrhythmias, myocarditis, MACE, and cardiac death were rare, each occurring in <1% of treated patients, with narrow CIs. Treatment discontinuation due to cardiac toxicity occurred in 0.54% (95% CI, 0.36% to 0.81%), and composite cardiac endpoints in 0.73% (95% CI, 0.50% to 1.06%). Individual outcome definitions, incidence estimates, and quality of evidence ratings are presented in Table 2.

**Table 2:** Summary of Pooled incidence and Quality of evidence of each study outcome.

Outcome	No. of Studies	Patients	Pooled Incidence (%; 95% CI)	Definitions Used in Studies	GRADE Certainty
LVEF Decline	50	1009	4.68% (3.35–6.52)	• Clinically significant LVEF decline (≥10% to <50%) • Grade ≥2 or ≥3 LVEF decline • LVEF decline ≥10–15% • Protocol-defined LVEF decline (<50% and ≥10–15% decrease) • LVEF≤40 • LVEF decline ≥20% • LV systolic dysfunction • Asymptomatic LVSD • LV dysfunction AE • Median change in LVEF • LVEF decline (CTCAE-defined)	**⊕⊕⊕ Moderate** (inconsistency due to variable LVEF definitions; risk of bias not downgraded because only 1 RCT and 2 single-arm studies were high risk)
Symptomatic HF	29	57	0.62% (0.43–0.88)	• NYHA class changes • Symptomatic HF • Cardiac failure • Symptomatic LVSD • HF grade ≥3 • HF requiring treatment • Chronic cardiac failure	**⊕⊕ Low** (very low event rate, serious imprecision, inconsistent reporting)
QTc Prolongation	5	23	0.37% (0.20–0.67)	• QTc_gt450 ms • QTc_ge480 ms • QTc_ge500 ms • ΔQTc >30 ms • Mean ΔΔQTcF values • QTc prolongation (any grade) • Grade ≥3 QTc prolongation • ECG abnormalities (QT-related)	**⊕⊕ Low** (very low event rate, heterogeneous QTc thresholds)
Arrhythmias	6	12	0.35% (0.23–0.54)	• Arrhythmia • Tachycardia • Supraventricular tachycardia • Treatment-related arrhythmia • Grade ≥3 arrhythmia	**⊕ Very Low** (extremely low event rate, very serious imprecision)
MACE	2	6	0.33% (0.24–0.46)	• Acute MI • Ischemic heart disease • Myocardial infarction • Angina • Cardiac arrest (non-fatal)	**⊕ Very Low** (low event rate, high imprecision)
Cardiac Death	18	12	0.35% (0.26–0.47)	• Cardiac death • Fatal MI • HF-related death • Cardiac arrest death • CHF-related multi-organ failure	**⊕ Very Low** (almost all zero events, serious imprecision)
Composite Cardiac Endpoint	13	291	0.73% (0.50–1.06)	• Primary cardiac endpoint • Adjudicated cardiac events • Primary/secondary cardiac event • Grade ≥3 cardiac events (composite) • Cardiac events (grade ≥3, composite) • Any cardiac AE • Cardiac AEs (trial-defined composite)	**⊕⊕⊕ Moderate** (larger sample, but heterogeneous composite definitions)
Treatment Discontinuation	9	168	0.54% (0.36–0.81)	• Discontinuation due to LVEF decline • Discontinuation due to cardiac toxicity • Arrhythmia leading to discontinuation	**⊕⊕ Low** (low event rates; inconsistent discontinuation criteria)

GRADE Working Group grades of evidence. High certainty: We are very confident that the true effect lies close to that of the estimate of the effect. Moderate certainty: We are moderately confident in the effect estimate: The true effect is likely to be close to the estimate of the effect, but there is a possibility it is substantially different. Low certainty: Our confidence in the effect estimate is limited: The true effect may be substantially different from the estimate of effect. Very low certainty: We have very little confidence in the effect estimate: The true effect is likely to be substantially different from the estimate of effect.

#### 3.1.3 Comparative risk of cardiotoxicity

**Fig. 4:**
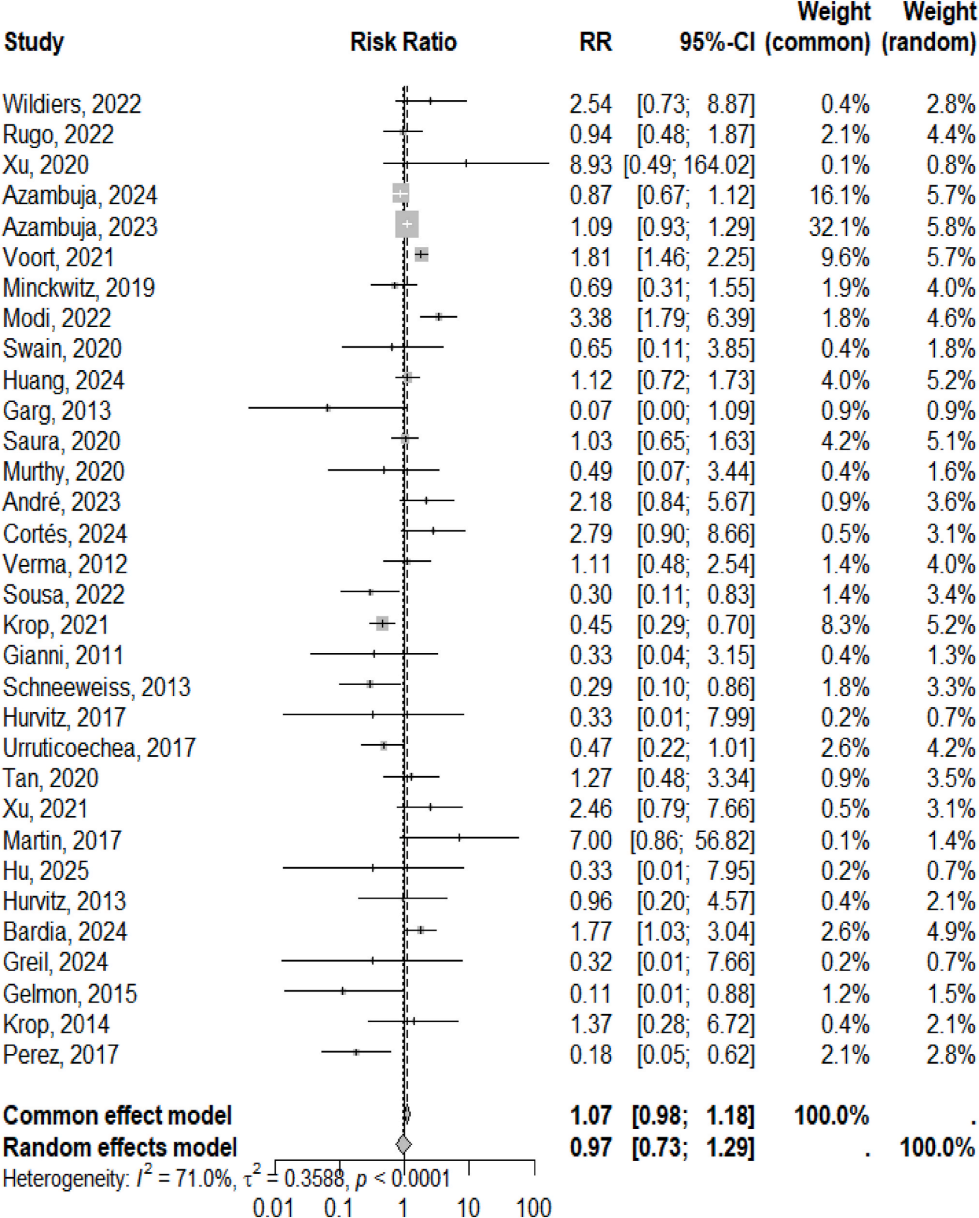
Forest plot of comparative risk of cardiotoxicity between newer HER2-directed therapies and conventional trastuzumab-based comparator regimens. The forest plot presents pooled risk ratios (RRs) comparing cardiotoxicity in patients receiving newer HER2-directed therapies versus conventional trastuzumab-based comparator regimens (including trastuzumab monotherapy or trastuzumab combined with chemotherapy, with or without pertuzumab). An RR > 1 indicates higher risk with newer therapies, while RR < 1 indicates lower risk. Squares represent individual study estimates, and the diamond represents the pooled estimate using a random-effects model. CI, confidence interval, RR, risk ratio.

From the eligible 33 RCTs, comparative data of 32 RCTs, including 27,895 patients reporting 1621 cardiotoxicity events, were analysed. The PHranceSCa trial was excluded from the comparative pooling because cardiac outcomes were reported only for the overall cohort (n = 160), without arm-specific data [41]. Overall, no significant difference in cardiotoxicity risk was observed between newer HER2-directed therapies and conventional trastuzumab-based comparator regimens (including trastuzumab monotherapy or trastuzumab combined with chemotherapy, with or without pertuzumab) (RR, 0.97; 95% CI, 0.73-1.29; p = 0.82), and heterogeneity was moderate (I^2^ = 71.0% [58.7–79.7%]; τ^2^ = 0.36) (Fig. 4; Table 3). The funnel plot showed mild asymmetry with Egger's test values as non-significant (t = −1.21; p = 0.236) (Supplementary Fig. 2).

**Table 3:** Comparative risk of cardiotoxicity between newer HER2-Directed therapies and conventional trastuzumab-based comparator regimens in randomized controlled trials.

Study	RR	95% CI	%W (Common)	%W (Random)
Wildiers, 2022	2.5366	[0.7250; 8.8747]	0.4	2.8
Rugo, 2022	0.9446	[0.4769; 1.8711]	2.1	4.4
Xu, 2020	8.9265	[0.4858; 164.0174]	0.1	0.8
Azambuja, 2024	0.8669	[0.6727; 1.1173]	16.1	5.7
Azambuja, 2023	1.0921	[0.9264; 1.2874]	32.1	5.8
Voort, 2021	1.8138	[1.4598; 2.2537]	9.6	5.7
Minckwitz, 2019	0.6950	[0.3107; 1.5544]	1.9	4.0
Modi, 2022	3.3844	[1.7923; 6.3907]	1.8	4.6
Swain, 2020	0.6471	[0.1087; 3.8517]	0.4	1.8
Huang, 2024	1.1189	[0.7237; 1.7297]	4.0	5.2
Garg, 2013	0.0657	[0.0040; 1.0851]	0.9	0.9
Saura, 2020	1.0264	[0.6455; 1.6320]	4.2	5.1
Murthy, 2020	0.4876	[0.0692; 3.4361]	0.4	1.6
André, 2023	2.1822	[0.8399; 5.6698]	0.9	3.6
Cortés, 2024	2.7928	[0.9010; 8.6571]	0.5	3.1
Verma, 2012	1.1102	[0.4845; 2.5441]	1.4	4.0
Sousa, 2022	0.2977	[0.1066; 0.8309]	1.4	3.4
Krop, 2021	0.4513	[0.2919; 0.6977]	8.3	5.2
Gianni, 2011	0.3333	[0.0352; 3.1539]	0.4	1.3
Schneeweiss, 2013	0.2941	[0.1011; 0.8556]	1.8	3.3
Hurvitz, 2017	0.3274	[0.0134; 7.9926]	0.2	0.7
Urruticoechea, 2017	0.4706	[0.2191; 1.0109]	2.6	4.2
Tan, 2020	1.2653	[0.4787; 3.3446]	0.9	3.5
Xu, 2021	2.4627	[0.7921; 7.6568]	0.5	3.1
Martin, 2017	7.0000	[0.8624; 56.8205]	0.1	1.4
Hu, 2025	0.3258	[0.0133; 7.9530]	0.2	0.7
Hurvitz, 2013	0.9565	[0.2001; 4.5716]	0.4	2.1
Bardia, 2024	1.7699	[1.0293; 3.0436]	2.6	4.9
Greil, 2024	0.3168	[0.0131; 7.6576]	0.2	0.7
Gelmon, 2015	0.1121	[0.0143; 0.8801]	1.2	1.5
Krop, 2014	1.3697	[0.2791; 6.7220]	0.4	2.1
Perez, 2017	0.1833	[0.0539; 0.6237]	2.1	2.8

CI, confidence interval; RR, risk ratio; W, weigh.

#### 3.1.4 Subgroup analysis

##### 3.1.4.1 Age

Across the age subgroups, cardiotoxicity estimates were broadly comparable. Studies with a median age ≥55 years had an incidence of 5.31% (95% CI, 2.85–9.69%), which is almost identical to those enrolling younger populations (<55 years: 5.26%; 95% CI, 3.03–8.97%). Both comparisons were marked by a very high heterogeneity (I^2^ > 93%), and no statistically significant difference was observed (P = 0.98) (Supplementary Fig. 3; Supplementary Table 2). The outcome was not reported in five studies [27,38,48,63,67].

##### 3.1.4.2 Drug class

There was more variation in the studies when assessed by drug class. Monoclonal antibody regimens showed a higher pooled incidence (8.04%) than ADCs (3.18%) or TKIs (3.41%). The overall difference was not statistically significant (P = 0.065) and heterogeneity was very high (I^2^ consistently >90%) (Supplementary Fig. 4; Supplementary Table 3).

##### 3.1.4.3 Anthracycline use

Cardiotoxicity was 6.01% (95% CI, 3.61–9.86%) in studies without anthracyclines and 4.39% (95% CI, 2.28–8.27%) in those with anthracyclines. The heterogeneity remained pronounced in both the groups (I^2^ > 95%), and subgroup differences were not significant (P = 0.43) (Supplementary Fig. 5; Supplementary Table 4).

##### 3.1.4.4 HER2 status

HER2-positive studies showed a pooled incidence of 4.80% (95% CI, 3.14–7.28%), whereas HER2-low studies (although limited to two small reports) had a higher estimate of 12.84% (95% CI, 0.14–93.84%). The subgroup difference was statistically significant (P = 0.012), and heterogeneity was high (I^2^ > 95%) (Supplementary Fig. 6; Supplementary Table 5).

##### 3.1.4.5 Agent used

There was variability across the various HER-2 directed agents used in the treatment of breast cancer. Pertuzumab-based regimens had the highest pooled incidence (7.77%), T-DM1 the lowest (2.10%), and T-DXd an intermediate estimate (7.19%). The heterogeneity rates were significant (I^2^ > 85%), and the higher rates seen with pyrotinib and zanidatamab may be attributed to small sample sizes (P < 0.0001 for subgroup difference) (Supplementary Fig. 7; Supplementary Table 6).

##### 3.1.4.6 Type of therapy

With the type of therapy used, combination regimens reported higher cardiotoxicity than monotherapy (6.11% vs. 3.14%; P = 0.07), with considerable heterogeneity (I^2^ = 94.7%) in both groups (Supplementary Fig. 8; Supplementary Table 7).

##### 3.1.4.7 Cardiac monitoring frequency

Studies with more frequent cardiac monitoring (≤8–9 weeks) showed a pooled cardiotoxicity incidence of 8.15% (95% CI, 3.13–19.61%), compared to those with longer intervals (>9 weeks) showing 5.14% (95% CI, 3.26–8.01%). This difference was not statistically significant (P = 0.349), with substantial heterogeneity in both subgroups (I^2^ > 94%). Fifteen studies did not report monitoring schedules [29,37,40,44,48,57,[59], [60], [61], [62],^64^,[69], [70], [71],^75^] (Supplementary Fig. 9; Supplementary Table 8).

##### 3.1.4.8 Sensitivity analysis

Sensitivity data were available from 55 studies, comprising 21,901 patients, of whom 1536 experienced cardiotoxicities. After excluding the three high-risk-of-bias studies [48,64,75], the pooled incidence of cardiotoxicity under the random-effects model was 5.33% (95% CI, 3.53–7.98%; τ^2^ = 1.50), which was highly consistent with the primary pooled estimate. Also, the heterogeneity remained significant (I^2^ = 95.8%) (Supplementary Fig. 10).

## 4 Discussion

In this study, we conducted a comprehensive systematic review and meta-analysis to quantify the incidence and comparative risk of cardiotoxicity associated with newer HER2-directed therapies compared with conventional trastuzumab-based comparator regimens (including trastuzumab monotherapy or trastuzumab combined with chemotherapy, with or without pertuzumab) across RCTs and prospective studies. Using all available cardiotoxicity data, we applied a random-effects logistic modelling framework that produced almost stable estimates. This method yielded results comparable to patient-level analyses, without the need for raw datasets. The clinical significance of this work is grounded in long-standing evidence that HER2-directed therapies have a measurable risk of cardiac dysfunction, which was first identified in pivotal trastuzumab trials [4,76] and later supported by large observational studies [77,78]. Building on this foundation, our work integrates a broader and more contemporary evidence base, thereby providing an updated and clinically meaningful assessment of cardiotoxicity across the expanding landscape of HER2-directed treatment options.

In a pooled analysis of more than 22,000 patients treated with newer HER2-directed therapies, the overall incidence of cardiotoxicity was approximately 5%, with clinically significant events occurring in less than 1% of the patients. These findings are lower than those reported with early trials of trastuzumab, where symptomatic cardiac dysfunction rates were 3-7% with monotherapy use and up to 27% when combined with anthracycline-based chemotherapy [4,79]. Similar findings are observed with real-world studies, such as the OHERA study, which reported the incidence of LV dysfunction in 7.6% of patients and congestive heart failure (CHF) in 2.8% of patients [80]. The overall pooled RRs of cardiotoxicity (RR 0.97; 95% CI 0.73–1.29) did not show a statistically significant increase in cardiotoxicity between newer HER2-directed therapies and comparator regimens. However, wide heterogeneity and differences in cardiac monitoring and study designs limit causal interpretation. While relative risks were comparable, the overall incidence of cardiotoxicity was lower than that reported in historical trastuzumab-based studies, suggesting an improved cardiac safety profile [81].

Subgroup analyses in our study further highlighted the complexity of cardiotoxicity risk in newer HER2-directed therapy. Cardiotoxicity rates were broadly consistent across age groups and treatment settings. When stratified by HER2 status, studies involving HER2-low populations showed a higher pooled incidence of cardiotoxicity compared with HER2-positive cohorts (12.8% vs 4.8%), although this estimate was based on only two studies with wide confidence intervals. This likely reflects differences in treatment patterns and sample size rather than a true biological effect.

Studies including patients with prior anthracycline exposure showed a slightly lower incidence of cardiotoxicity. This may be explained by selection bias, as patients with preserved cardiac function are more likely to receive subsequent HER2-directed therapies. It may also reflect improved clinical practice, including careful patient selection, dose optimisation, and enhanced cardiac monitoring. This contrasts with earlier findings from NSABP-B31, HERA, BCIRG-006, and real-world cohorts, where older age and prior anthracycline therapy were strong predictors of LV dysfunction and heart failure [[82], [83], [84]].

In our analysis, differences were observed across drug classes, individual agents, and treatment strategies. However, most of these findings were not statistically significant and should be interpreted with caution due to high heterogeneity and limited data in several subgroups. Monoclonal antibody-based regimens, including pertuzumab-containing therapies, showed higher cardiotoxicity estimates in some studies compared with ADCs and TKIs, but these comparisons were not statistically significant. Similarly, combination regimens showed numerically higher cardiotoxicity rates than monotherapy, but the findings were not statistically significant.

An important observation from this analysis is the influence of cardiac monitoring practices. Studies with more frequent cardiac monitoring reported higher cardiotoxicity events, indicating the need for standardized and consistent surveillance practices in future research. In the current therapeutic era, variations in molecular design, mechanism of action, and regimen complexity may have a greater impact on cardiotoxicity risk than conventional patient-level predictors.

To the best of our knowledge, this study provides one of the most comprehensive assessments of cardiotoxicity associated with newer HER2-directed therapies used beyond trastuzumab monotherapy. Because these agents are now widely used in both early-stage and metastatic disease, our findings have clear, real-world relevance. The pooled incidence estimates and comparative risks provide clinicians with a more accurate picture of the absolute and relative cardiac risks. This data can support more informed treatment planning and more balanced conversations with patients. Although severe cardiac events were uncommon, our results emphasize the importance of coordinated care involving oncology, cardiology, and dedicated cardio-oncology teams to enable early detection and prompt management of LVEF decline, arrhythmias, QTc prolongation, and other cardiac toxicities.

A major strength of this study is its broad and methodologically rigorous assessment of cardiotoxicity across a wide range of HER2-directed therapies. By bringing together evidence from both RCTs and non-randomized prospective studies, we were able to identify meaningful patterns of cardiac risk that would not be visible in individual trials alone. We also conducted extensive subgroup analyses, assessing factors such as age, HER2 status, anthracycline exposure, agent class, treatment setting, and cardiac monitoring frequency. This allowed us to explore how both patient characteristics and treatment choices influence cardiac risk. Hence, these findings will strengthen the real-world data and support more tailored patient counselling, risk stratification, and monitoring strategies in everyday practice.

This study also has several limitations that should be considered when interpreting the results. There was considerable heterogeneity across the analyses, likely due to the differences in the study populations, drug regimens, cardiac monitoring practices, and definitions of cardiotoxicity that could not be fully accounted for. Although our sensitivity analyses showed stable estimates, the possibility of unmeasured confounding cannot be ruled out. Reporting of cardiac outcomes was also variable with many studies not using standardized criteria for LVEF decline, symptomatic heart failure, or arrhythmias, and several trials did not report certain outcomes, which limited the accuracy of some subgroup comparisons. Most studies were open-label, introducing potential reporting bias, especially for clinically adjudicated outcomes. Finally, because our analysis is based on study-level rather than individual patient data, we could not investigate important modifiers such as baseline cardiac function, cumulative dose, or comorbidities. Future research using harmonized cardiac definitions and individual patient data will be essential to refine these estimates further.

## 5 Conclusion

Our findings indicate that cardiotoxicity with newer HER2-directed therapies remains infrequent and generally manageable, with clear variation across individual agents and treatment settings. Comparative analyses suggest no significant increase in the risk of cardiotoxicity associated with newer HER2-directed therapies versus conventional trastuzumab-based regimens (including trastuzumab monotherapy or trastuzumab combined with chemotherapy, with or without pertuzumab). These findings support the continued use of these therapies with appropriate cardiac monitoring. As HER2-directed treatments continue to evolve, integrating structured cardiac surveillance and individualized risk assessment will be essential to optimize patient safety while preserving treatment benefit.

## Data availability

All data used in this systematic review and meta-analysis were obtained from previously published studies. No individual patient-level data were accessed. The extracted datasets and analysis files are available from the corresponding author upon reasonable request.

## Copyright and originality declaration

All authors confirm that:

• This manuscript is original, has not been published previously, and is not under consideration elsewhere.
• All authors meet the criteria for authorship and have approved the final version of the manuscript.
• The work is free from plagiarism, fabrication, and falsification.
• The corresponding author will act on behalf of all authors during the peer-review process and post-publication.

## Declaration of generative AI and AI-assisted technologies in the manuscript preparation process

The authors declare that generative AI and AI-assisted technologies were used solely for language refinement and grammatical editing during the manuscript preparation. No AI tools were used for data analysis, interpretation, or generation of scientific content. All authors take full responsibility for the accuracy and integrity of the work**.**

## Funding

This research did not receive any specific grant from funding agencies in the public, commercial, or not-for-profit sectors.

## CRediT authorship contribution statement

**Senthamizhan Sundaramoorthy:** Conceptualization, Data curation, Methodology, Project administration, Writing – original draft, Writing – review & editing. **Narendhar Gokulanathan:** Conceptualization, Data curation, Methodology, Project administration, Validation. **Rona Joseph P:** Conceptualization, Methodology, Project administration, Supervision. **Anoop Tm:** Conceptualization, Methodology, Project administration, Supervision. **Sherin P. Mathew:** Data curation, Investigation, Supervision. **Harpreet Kaur:** Formal analysis, Software. **Thamizharuvi Muthukumarasamy:** Formal analysis, Software. **Joe Jose:** Data curation, Investigation. **Kalai Bharathi Jayaraman:** Data curation, Investigation. **Parvathy Rajmohan:** Data curation, Investigation. **Vyshak Thovarayi:** Data curation, Investigation. **Aswin Sathyadas:** Data curation, Investigation. **Shibu V. Ignatious:** Data curation, Investigation. **Sariga Ajit Valaparambil:** Data curation, Investigation.

## Declaration of competing interests

The authors declare that they have no conflicting interests.
