# Supplementary material for: Cardiotoxicity of newer HER2-Directed therapies in breast cancer: A systematic review and meta-analysis of incidence and comparative risk
Source: Breast. 2026 Jul 20;89:104880. doi: 10.1016/j.breast.2026.104880 (PMC13446359; doi:10.1016/j.breast.2026.104880)
Supplement: Supplementary file 1 [file mmc1.docx]

**Supplementary File**

Search Strategy

1. Cochrane Central

Access date: 12 September 2025

Search strategy:

1	MeSH descriptor: [Breast Neoplasms] explode all trees	21246

2	(breast cancer OR breast neoplasm* OR mammary carcinoma* OR mammary neoplasm*):ti,ab,kw (Word variations have been searched)	51408

3	MeSH descriptor: [Receptor, ErbB-2] explode all trees	1775

4	(HER2 OR ERBB2 OR "human epidermal growth factor receptor 2" OR trastuzumab OR pertuzumab OR "trastuzumab deruxtecan" OR T-DXd OR "trastuzumab emtansine" OR T-DM1 OR kadcyla OR tucatinib OR neratinib OR margetuximab OR lapatinib OR pyrotinib):ti,ab,kw (Word variations have been searched)	10535

5	MeSH descriptor: [Cardiotoxicity] explode all trees	242

6	(cardiotoxic* OR (cardiac NEXT dysfunction) OR (left NEXT ventricular NEXT dysfunction) OR (LV NEXT dysfunction) OR (heart NEXT failure) OR (reduced NEXT ejection NEXT fraction) OR (cardiac NEXT event*) OR (cardiac NEXT adverse NEXT event*)):ti,ab,kw (Word variations have been searched)	49500

7	MeSH descriptor: [Heart Diseases] explode all trees	75995

8	#1 OR #2	51411

9	#3 OR #4	10708

10	#5 OR #6 OR #7	102925

11	(#1 OR #2) AND (#3 OR #4) AND (#5 OR #6 OR #7)	605

Search results: 605

2. PubMed

Access date: 12 September 2025

Search strategy: (("Breast Neoplasms"[Mesh] OR "breast cancer"[tiab] OR "breast neoplasm*"[tiab] OR "mammary carcinoma*"[tiab] OR "mammary neoplasm*"[tiab])) AND (("Receptor, ErbB-2"[Mesh] OR HER2[tiab] OR ERBB2[tiab] OR "human epidermal growth factor receptor 2"[tiab] OR trastuzumab[tiab] OR pertuzumab[tiab] OR "trastuzumab deruxtecan"[tiab] OR T-DXd[tiab] OR "trastuzumab emtansine"[tiab] OR T-DM1[tiab] OR kadcyla[tiab] OR tucatinib[tiab] OR neratinib[tiab] OR pyrotinib[tiab] OR poziotinib[tiab] OR margetuximab[tiab] OR zanidatamab[tiab] OR "trastuzumab duocarmazine"[tiab] OR SYD985[tiab] OR RC48[tiab] OR ARX788[tiab] OR lapatinib[tiab]))AND (("Cardiotoxicity"[Mesh] OR "Heart Diseases"[Mesh] OR cardiotoxic*[tiab] OR "cardiac dysfunction"[tiab] OR "left ventricular dysfunction"[tiab] OR "LV dysfunction"[tiab] OR "heart failure"[tiab] OR "reduced ejection fraction"[tiab] OR "cardiac event*"[tiab] OR "cardiac adverse event*"[tiab]))

Search results: 1741 results

3. Scopus

Access date: 13 September 2025

Search strategy: TITLE-ABS-KEY("breast cancer" OR "breast neoplasm*" OR "mammary carcinoma*") AND TITLE-ABS-KEY(HER2 OR ERBB2 OR "human epidermal growth factor receptor 2" OR trastuzumab OR pertuzumab OR "trastuzumab deruxtecan" OR T-DXd OR "trastuzumab emtansine" OR T-DM1 OR tucatinib OR neratinib OR pyrotinib OR poziotinib OR margetuximab OR zanidatamab OR "trastuzumab duocarmazine" OR SYD985 OR RC48 OR ARX788 OR lapatinib) AND TITLE-ABS-KEY(cardiotoxic* OR "cardiac dysfunction" OR "heart failure" OR "left ventricular dysfunction" OR "reduced ejection fraction")

Search results: 305

4. Embase

Access date: 13 September 2025

Search strategy: Emtree("breast tumor")/exp OR (breast NEAR/1 cancer):ti,ab,kw OR (breast neoplasm*):ti,ab,kw OR (mammary carcinoma*):ti,ab,kw Emtree("erbB-2 receptor")/exp OR HER2:ti,ab,kw OR ERBB2:ti,ab,kw OR "human epidermal growth factor receptor 2":ti,ab,kw OR trastuzumab:ti,ab,kw OR pertuzumab:ti,ab,kw OR ("trastuzumab" NEAR/2 deruxtecan):ti,ab,kw OR T-DXd:ti,ab,kw OR ("trastuzumab" NEAR/2 emtansine):ti,ab,kw OR T-DM1:ti,ab,kw OR kadcyla:ti,ab,kw OR tucatinib:ti,ab,kw OR neratinib:ti,ab,kw OR pyrotinib:ti,ab,kw OR poziotinib:ti,ab,kw OR margetuximab:ti,ab,kw OR zanidatamab:ti,ab,kw OR ("trastuzumab" NEAR/2 duocarmazine):ti,ab,kw OR SYD985:ti,ab,kw OR RC48:ti,ab,kw OR ARX788:ti,ab,kw OR lapatinib:ti,ab,kw mtree("cardiotoxicity")/exp OR Emtree("heart disease")/exp OR cardiotoxic*:ti,ab,kw OR (cardiac NEAR/3 dysfunction):ti,ab,kw OR (left NEAR/2 ventricular NEAR/2 dysfunction):ti,ab,kw OR (heart NEAR/1 failure):ti,ab,kw OR ("reduced" NEAR/2 "ejection" NEAR/2 fraction):ti,ab,kw

Search results: 1454

**Supplementary Table1. Pooled incidence of Cardiotoxicity**

| Study | Proportion | 95%-CI |
| --- | --- | --- |
| Wildiers, 2022 | 0.1951 | [0.0882; 0.3487] |
| Rugo, 2022 | 0.0568 | [0.0321; 0.0920] |
| Yang, 2024 | 0.0833 | [0.0367; 0.1576] |
| Portera, 2008 | 0.5455 | [0.2338; 0.8325] |
| Xu, 2020 | 0.0328 | [0.0090; 0.0818] |
| Yamashita, 2021 | 0.0000 | [0.0000; 0.0725] |
| Gavilá, 2019 | 0.0241 | [0.0029; 0.0843] |
| Levicki, 2023 | 0.0769 | [0.0095; 0.2513] |
| Azambuja, 2024 | 0.0509 | [0.0419; 0.0613] |
| Azambuja, 2023 | 0.1113 | [0.0989; 0.1246] |
| Morris, 2013 | 0.0303 | [0.0099; 0.0693] |
| Dang, 2022 | 0.2191 | [0.1794; 0.2631] |
| Gherghe, 2022 | 0.2273 | [0.0782; 0.4537] |
| Tian, 2024 | 0.0680 | [0.0278; 0.1350] |
| Wang, 2022 | 0.4091 | [0.2071; 0.6365] |
| Yu, 2023 | 0.1408 | [0.0697; 0.2438] |
| Voort, 2021 | 0.6018 | [0.5340; 0.6669] |
| Krop, 2015 | 0.0338 | [0.0111; 0.0771] |
| Minckwitz, 2019 | 0.0135 | [0.0065; 0.0247] |
| Lenihan, 2011 | 0.1178 | [0.0851; 0.1575] |
| Modi, 2022 | 0.1968 | [0.1575; 0.2409] |
| Ferre, 2020 | 0.1333 | [0.0376; 0.3072] |
| Yuan, 2021 | 0.0400 | [0.0010; 0.2035] |
| Khoury, 2021 | 0.6333 | [0.4386; 0.8007] |
| Perez, 2008 | 0.0266 | [0.0215; 0.0324] |
| Swain, 2020 | 0.0049 | [0.0006; 0.0176] |
| Romani, 2025 | 0.1961 | [0.0982; 0.3312] |
| Huang, 2024 | 0.2339 | [0.1794; 0.2959] |
| Garg, 2013 | 0.0000 | [0.0000; 0.1684] |
| Perez, 2016 | 0.1887 | [0.1192; 0.2762] |
| Kuemmel, 2021 | 0.1068 | [0.0787; 0.1407] |
| Cheng, 2023 | 0.2917 | [0.1262; 0.5109] |
| Modi, 2019 | 0.0163 | [0.0034; 0.0469] |
| Saura, 2020 | 0.1056 | [0.0734; 0.1458] |
| Murthy, 2020 | 0.0050 | [0.0006; 0.0178] |
| André, 2023 | 0.0593 | [0.0375; 0.0884] |
| Cortés, 2024 | 0.0428 | [0.0216; 0.0753] |
| Harbeck, 2024 | 0.1270 | [0.0992; 0.1592] |
| Verma, 2012 | 0.0249 | [0.0130; 0.0432] |
| Sousa, 2022 | 0.0183 | [0.0074; 0.0373] |
| Krop, 2021 | 0.0307 | [0.0205; 0.0441] |
| Gianni, 2011 | 0.0093 | [0.0002; 0.0510] |
| Schneeweiss, 2013 | 0.0588 | [0.0163; 0.1438] |
| Hurvitz, 2017 | 0.0000 | [0.0000; 0.0164] |
| Miles, 2021 | 0.1643 | [0.1455; 0.1845] |
| Urruticoechea, 2017 | 0.0877 | [0.0544; 0.1322] |
| Shaughnessy, 2021 | 0.0625 | [0.0304; 0.1119] |
| Tan, 2020 | 0.0357 | [0.0165; 0.0667] |
| Xu, 2021 | 0.0746 | [0.0364; 0.1330] |
| Martin, 2017 | 0.0050 | [0.0020; 0.0102] |
| Hu, 2025 | 0.0000 | [0.0000; 0.0166] |
| Hurvitz, 2013 | 0.0435 | [0.0091; 0.1218] |
| Bardia, 2024 | 0.0806 | [0.0568; 0.1104] |
| Michalaki, 2017 | 0.0000 | [0.0000; 0.1277] |
| Greil, 2024 | 0.0000 | [0.0000; 0.0451] |
| Gelmon, 2015 | 0.0031 | [0.0001; 0.0172] |
| Krop, 2014 | 0.0149 | [0.0055; 0.0321] |
| Perez, 2017 | 0.0083 | [0.0017; 0.0241] |

Supplementary Table 2. Subgroup analysis by age

| Study | Proportion | 95% CI | Age_Median |
| --- | --- | --- | --- |
| Wildiers, 2022 | 0.1951 | [0.0882; 0.3487] | ≥55 |
| Rugo, 2022 | 0.0568 | [0.0321; 0.0920] | ≥55 |
| Yang, 2024 | 0.0833 | [0.0367; 0.1576] | <55 |
| Portera, 2008 | 0.5455 | [0.2338; 0.8325] | <55 |
| Xu, 2020 | 0.0328 | [0.0090; 0.0818] | <55 |
| Yamashita, 2021 | 0.0000 | [0.0000; 0.0725] | ≥55 |
| Gavilá, 2019 | 0.0241 | [0.0029; 0.0843] | <55 |
| Levicki, 2023 | 0.0769 | [0.0095; 0.2513] | ≥55 |
| Azambuja, 2024 | 0.0509 | [0.0419; 0.0613] | <55 |
| Azambuja, 2023 | 0.1113 | [0.0989; 0.1246] | <55 |
| Morris, 2013 | 0.0303 | [0.0099; 0.0693] | <55 |
| Dang, 2022 | 0.2191 | [0.1794; 0.2631] | <55 |
| Gherghe, 2022 | 0.2273 | [0.0782; 0.4537] | ≥55 |
| Tian, 2024 | 0.0680 | [0.0278; 0.1350] | <55 |
| Wang, 2022 | 0.4091 | [0.2071; 0.6365] | ≥55 |
| Yu, 2023 | 0.1408 | [0.0697; 0.2438] | <55 |
| Voort, 2021 | 0.6018 | [0.5340; 0.6669] | <55 |
| Minckwitz, 2019 | 0.0135 | [0.0065; 0.0247] | <55 |
| Lenihan, 2011 | 0.1178 | [0.0851; 0.1575] | ≥55 |
| Modi, 2022 | 0.1968 | [0.1575; 0.2409] | ≥55 |
| Ferre, 2020 | 0.1333 | [0.0376; 0.3072] | <55 |
| Yuan, 2021 | 0.0400 | [0.0010; 0.2035] | ≥55 |
| Perez, 2008 | 0.0266 | [0.0215; 0.0324] | ≥55 |
| Romani, 2025 | 0.1961 | [0.0982; 0.3312] | <55 |
| Huang, 2024 | 0.2339 | [0.1794; 0.2959] | <55 |
| Garg, 2013 | 0.0000 | [0.0000; 0.1684] | <55 |
| Perez, 2016 | 0.1887 | [0.1192; 0.2762] | ≥55 |
| Kuemmel, 2021 | 0.1068 | [0.0787; 0.1407] | ≥55 |
| Cheng, 2023 | 0.2917 | [0.1262; 0.5109] | <55 |
| Modi, 2019 | 0.0163 | [0.0034; 0.0469] | ≥55 |
| Saura, 2020 | 0.1056 | [0.0734; 0.1458] | ≥55 |
| Murthy, 2020 | 0.0050 | [0.0006; 0.0178] | ≥55 |
| André, 2023 | 0.0593 | [0.0375; 0.0884] | <55 |
| Cortés, 2024 | 0.0428 | [0.0216; 0.0753] | <55 |
| Harbeck, 2024 | 0.1270 | [0.0992; 0.1592] | <55 |
| Verma, 2012 | 0.0249 | [0.0130; 0.0432] | <55 |
| Sousa, 2022 | 0.0183 | [0.0074; 0.0373] | ≥55 |
| Krop, 2021 | 0.0307 | [0.0205; 0.0441] | <55 |
| Gianni, 2011 | 0.0093 | [0.0002; 0.0510] | <55 |
| Hurvitz, 2017 | 0.0000 | [0.0000; 0.0164] | <55 |
| Miles, 2021 | 0.1643 | [0.1455; 0.1845] | <55 |
| Urruticoechea, 2017 | 0.0877 | [0.0544; 0.1322] | ≥55 |
| Shaughnessy, 2021 | 0.0625 | [0.0304; 0.1119] | <55 |
| Tan, 2020 | 0.0357 | [0.0165; 0.0667] | <55 |
| Xu, 2021 | 0.0746 | [0.0364; 0.1330] | <55 |
| Martin, 2017 | 0.0050 | [0.0020; 0.0102] | <55 |
| Hu, 2025 | 0.0000 | [0.0000; 0.0166] | <55 |
| Hurvitz, 2013 | 0.0435 | [0.0091; 0.1218] | ≥55 |
| Bardia, 2024 | 0.0806 | [0.0568; 0.1104] | ≥55 |
| Michalaki, 2017 | 0.0000 | [0.0000; 0.1277] | <55 |
| Gelmon, 2015 | 0.0031 | [0.0001; 0.0172] | ≥55 |
| Krop, 2014 | 0.0149 | [0.0055; 0.0321] | <55 |
| Perez, 2017 | 0.0083 | [0.0017; 0.0241] | ≥55 |

CI, confidence interval

Supplementary Table 3. Subgroup analysis by drug class

| Study | Proportion | 95% CI | Drug Class |
| --- | --- | --- | --- |
| Wildiers, 2022 | 0.1951 | [0.0882, 0.3487] | Monoclonal antibody |
| Rugo, 2022 | 0.0568 | [0.0321, 0.0920] | Monoclonal antibody |
| Yang, 2024 | 0.0833 | [0.0367, 0.1576] | Monoclonal antibody |
| Portera, 2008 | 0.5455 | [0.2338, 0.8325] | Monoclonal antibody |
| Xu, 2020 | 0.0328 | [0.0090, 0.0818] | Monoclonal antibody |
| Yamashita, 2021 | 0.0000 | [0.0000, 0.0725] | Monoclonal antibody |
| Gavilá, 2019 | 0.0241 | [0.0029, 0.0843] | Monoclonal antibody |
| Levicki, 2023 | 0.0769 | [0.0095, 0.2513] | Antibody–drug conjugate |
| Azambuja, 2024 | 0.0509 | [0.0419, 0.0613] | Tyrosine kinase inhibitor |
| Azambuja, 2023 | 0.1113 | [0.0989, 0.1246] | Monoclonal antibody |
| Morris, 2013 | 0.0303 | [0.0099, 0.0693] | Tyrosine kinase inhibitor |
| Dang, 2022 | 0.2191 | [0.1794, 0.2631] | Monoclonal antibody |
| Gherghe, 2022 | 0.2273 | [0.0782, 0.4537] | Monoclonal antibody |
| Tian, 2024 | 0.0680 | [0.0278, 0.1350] | Monoclonal antibody |
| Wang, 2022 | 0.4091 | [0.2071, 0.6365] | Tyrosine kinase inhibitor |
| Yu, 2023 | 0.1408 | [0.0697, 0.2438] | Monoclonal antibody |
| Voort, 2021 | 0.6018 | [0.5340, 0.6669] | Monoclonal antibody |
| Krop, 2015 | 0.0338 | [0.0111, 0.0771] | Antibody–drug conjugate |
| Minckwitz, 2019 | 0.0135 | [0.0065, 0.0247] | Antibody–drug conjugate |
| Lenihan, 2011 | 0.1178 | [0.0851, 0.1575] | Monoclonal antibody |
| Modi, 2022 | 0.1968 | [0.1575, 0.2409] | Antibody–drug conjugate |
| Ferre, 2020 | 0.1333 | [0.0376, 0.3072] | Tyrosine kinase inhibitor |
| Yuan, 2021 | 0.0400 | [0.0010, 0.2035] | Tyrosine kinase inhibitor |
| Khoury, 2021 | 0.6333 | [0.4386, 0.8007] | Monoclonal antibody + ADC |
| Perez, 2008 | 0.0266 | [0.0215, 0.0324] | Tyrosine kinase inhibitor |
| Swain, 2020 | 0.0049 | [0.0006, 0.0176] | Monoclonal antibody |
| Romani, 2025 | 0.1961 | [0.0982, 0.3312] | Monoclonal antibody |
| Huang, 2024 | 0.2339 | [0.1794, 0.2959] | Monoclonal antibody |
| Garg, 2013 | 0.0000 | [0.0000, 0.1684] | Monoclonal antibody |
| Perez, 2016 | 0.1887 | [0.1192, 0.2762] | Monoclonal antibody |
| Kuemmel, 2021 | 0.1068 | [0.0787, 0.1407] | Monoclonal antibody |
| Cheng, 2023 | 0.2917 | [0.1262, 0.5109] | Monoclonal antibody |
| Modi, 2019 | 0.0163 | [0.0034, 0.0469] | Antibody–drug conjugate |
| Saura, 2020 | 0.1056 | [0.0734, 0.1458] | Tyrosine kinase inhibitor |
| Murthy, 2020 | 0.0050 | [0.0006, 0.0178] | Tyrosine kinase inhibitor |
| André, 2023 | 0.0593 | [0.0375, 0.0884] | Antibody–drug conjugate |
| Cortés, 2024 | 0.0428 | [0.0216, 0.0753] | Antibody–drug conjugate |
| Harbeck, 2024 | 0.1270 | [0.0992, 0.1592] | Antibody–drug conjugate |
| Verma, 2012 | 0.0249 | [0.0130, 0.0432] | Antibody–drug conjugate |
| Sousa, 2022 | 0.0183 | [0.0074, 0.0373] | Antibody–drug conjugate |
| Krop, 2021 | 0.0307 | [0.0205, 0.0441] | Antibody–drug conjugate |
| Gianni, 2011 | 0.0093 | [0.0002, 0.0510] | Monoclonal antibody |
| Schneeweiss, 2013 | 0.0588 | [0.0163, 0.1438] | Monoclonal antibody |
| Hurvitz, 2017 | 0.0000 | [0.0000, 0.0164] | Monoclonal antibody + ADC |
| Miles, 2021 | 0.1643 | [0.1455, 0.1845] | Monoclonal antibody |
| Urruticoechea, 2017 | 0.0877 | [0.0544, 0.1322] | Monoclonal antibody |
| Shaughnessy, 2021 | 0.0625 | [0.0304, 0.1119] | Monoclonal antibody |
| Tan, 2020 | 0.0357 | [0.0165, 0.0667] | Monoclonal antibody |
| Xu, 2021 | 0.0746 | [0.0364, 0.1330] | Tyrosine kinase inhibitor |
| Martin, 2017 | 0.0050 | [0.0020, 0.0102] | Tyrosine kinase inhibitor |
| Hu, 2025 | 0.0000 | [0.0000, 0.0166] | Antibody–drug conjugate |
| Hurvitz, 2013 | 0.0435 | [0.0091, 0.1218] | Antibody–drug conjugate |
| Bardia, 2024 | 0.0806 | [0.0568, 0.1104] | Antibody–drug conjugate |
| Michalaki, 2017 | 0.0000 | [0.0000, 0.1277] | Monoclonal antibody |
| Greil, 2024 | 0.0000 | [0.0000, 0.0451] | Monoclonal antibody |
| Gelmon, 2015 | 0.0031 | [0.0001, 0.0172] | Tyrosine kinase inhibitor |
| Krop, 2014 | 0.0149 | [0.0055, 0.0321] | Antibody–drug conjugate |
| Perez, 2017 | 0.0083 | [0.0017, 0.0241] | Antibody–drug conjugate |

CI, confidence interval

Supplementary Table 4. Subgroup analysis by anthracycline use

| Study | Proportion | 95% CI | Anthracycline Use |
| --- | --- | --- | --- |
| Wildiers, 2022 | 0.1951 | [0.0882, 0.3487] | No |
| Rugo, 2022 | 0.0568 | [0.0321, 0.0920] | Yes |
| Yang, 2024 | 0.0833 | [0.0367, 0.1576] | Yes |
| Portera, 2008 | 0.5455 | [0.2338, 0.8325] | Yes |
| Xu, 2020 | 0.0328 | [0.0090, 0.0818] | No |
| Yamashita, 2021 | 0.0000 | [0.0000, 0.0725] | Yes |
| Gavilá, 2019 | 0.0241 | [0.0029, 0.0843] | Yes |
| Levicki, 2023 | 0.0769 | [0.0095, 0.2513] | Yes |
| Azambuja, 2024 | 0.0509 | [0.0419, 0.0613] | Yes |
| Azambuja, 2023 | 0.1113 | [0.0989, 0.1246] | Yes |
| Morris, 2013 | 0.0303 | [0.0099, 0.0693] | Yes |
| Dang, 2022 | 0.2191 | [0.1794, 0.2631] | Yes |
| Gherghe, 2022 | 0.2273 | [0.0782, 0.4537] | No |
| Tian, 2024 | 0.0680 | [0.0278, 0.1350] | No |
| Wang, 2022 | 0.4091 | [0.2071, 0.6365] | Yes |
| Yu, 2023 | 0.1408 | [0.0697, 0.2438] | Yes |
| Voort, 2021 | 0.6018 | [0.5340, 0.6669] | Yes |
| Krop, 2015 | 0.0338 | [0.0111, 0.0771] | Yes |
| Minckwitz, 2019 | 0.0135 | [0.0065, 0.0247] | Yes |
| Lenihan, 2011 | 0.1178 | [0.0851, 0.1575] | No |
| Modi, 2022 | 0.1968 | [0.1575, 0.2409] | No |
| Ferre, 2020 | 0.1333 | [0.0376, 0.3072] | No |
| Yuan, 2021 | 0.0400 | [0.0010, 0.2035] | No |
| Khoury, 2021 | 0.6333 | [0.4386, 0.8007] | Yes |
| Perez, 2008 | 0.0266 | [0.0215, 0.0324] | Yes |
| Swain, 2020 | 0.0049 | [0.0006, 0.0176] | No |
| Romani, 2025 | 0.1961 | [0.0982, 0.3312] | No |
| Huang, 2024 | 0.2339 | [0.1794, 0.2959] | No |
| Garg, 2013 | 0.0000 | [0.0000, 0.1684] | No |
| Perez, 2016 | 0.1887 | [0.1192, 0.2762] | No |
| Kuemmel, 2021 | 0.1068 | [0.0787, 0.1407] | No |
| Cheng, 2023 | 0.2917 | [0.1262, 0.5109] | No |
| Modi, 2019 | 0.0163 | [0.0034, 0.0469] | No |
| Saura, 2020 | 0.1056 | [0.0734, 0.1458] | No |
| Murthy, 2020 | 0.0050 | [0.0006, 0.0178] | No |
| André, 2023 | 0.0593 | [0.0375, 0.0884] | No |
| Cortés, 2024 | 0.0428 | [0.0216, 0.0753] | No |
| Harbeck, 2024 | 0.1270 | [0.0992, 0.1592] | No |
| Verma, 2012 | 0.0249 | [0.0130, 0.0432] | Yes |
| Sousa, 2022 | 0.0183 | [0.0074, 0.0373] | No |
| Krop, 2021 | 0.0307 | [0.0205, 0.0441] | Yes |
| Gianni, 2011 | 0.0093 | [0.0002, 0.0510] | Yes |
| Schneeweiss, 2013 | 0.0588 | [0.0163, 0.1438] | Yes |
| Hurvitz, 2017 | 0.0000 | [0.0000, 0.0164] | No |
| Miles, 2021 | 0.1643 | [0.1455, 0.1845] | No |
| Urruticoechea, 2017 | 0.0877 | [0.0544, 0.1322] | Yes |
| Shaughnessy, 2021 | 0.0625 | [0.0304, 0.1119] | Yes |
| Tan, 2020 | 0.0357 | [0.0165, 0.0667] | Yes |
| Xu, 2021 | 0.0746 | [0.0364, 0.1330] | No |
| Martin, 2017 | 0.0050 | [0.0020, 0.0102] | Yes |
| Hu, 2025 | 0.0000 | [0.0000, 0.0166] | Yes |
| Hurvitz, 2013 | 0.0435 | [0.0091, 0.1218] | Yes |
| Bardia, 2024 | 0.0806 | [0.0568, 0.1104] | No |
| Michalaki, 2017 | 0.0000 | [0.0000, 0.1277] | No |
| Greil, 2024 | 0.0000 | [0.0000, 0.0451] | Yes |
| Gelmon, 2015 | 0.0031 | [0.0001, 0.0172] | Yes |
| Krop, 2014 | 0.0149 | [0.0055, 0.0321] | No |
| Perez, 2017 | 0.0083 | [0.0017, 0.0241] | Yes |

CI, confidence interval

Supplementary Table 5. Subgroup analysis by HER2 status

| Study | Proportion | 95% CI | HER2 Status |
| --- | --- | --- | --- |
| Wildiers, 2022 | 0.1951 | [0.0882; 0.3487] | Positive |
| Rugo, 2022 | 0.0568 | [0.0321; 0.0920] | Positive |
| Yang, 2024 | 0.0833 | [0.0367; 0.1576] | Positive |
| Portera, 2008 | 0.5455 | [0.2338; 0.8325] | Positive |
| Xu, 2020 | 0.0328 | [0.0090; 0.0818] | Positive |
| Yamashita, 2021 | 0.0 | [0.0000; 0.0725] | Positive |
| Gavilá, 2019 | 0.0241 | [0.0029; 0.0843] | Positive |
| Levicki, 2023 | 0.0769 | [0.0095; 0.2513] | Positive |
| Azambuja, 2024 | 0.0509 | [0.0419; 0.0613] | Positive |
| Azambuja, 2023 | 0.1113 | [0.0989; 0.1246] | Positive |
| Morris, 2013 | 0.0303 | [0.0099; 0.0693] | Positive |
| Dang, 2022 | 0.2191 | [0.1794; 0.2631] | Positive |
| Gherghe, 2022 | 0.2273 | [0.0782; 0.4537] | Positive |
| Tian, 2024 | 0.068 | [0.0278; 0.1350] | Positive |
| Wang, 2022 | 0.4091 | [0.2071; 0.6365] | Positive |
| Yu, 2023 | 0.1408 | [0.0697; 0.2438] | Positive |
| Voort, 2021 | 0.6018 | [0.5340; 0.6669] | Positive |
| Krop, 2015 | 0.0338 | [0.0111; 0.0771] | Positive |
| Minckwitz, 2019 | 0.0135 | [0.0065; 0.0247] | Positive |
| Lenihan, 2011 | 0.1178 | [0.0851; 0.1575] | Positive |
| Modi, 2022 | 0.1968 | [0.1575; 0.2409] | Low |
| Ferre, 2020 | 0.1333 | [0.0376; 0.3072] | Positive |
| Yuan, 2021 | 0.04 | [0.0010; 0.2035] | Positive |
| Khoury, 2021 | 0.6333 | [0.4386; 0.8007] | Positive |
| Perez, 2008 | 0.0266 | [0.0215; 0.0324] | Positive |
| Swain, 2020 | 0.0049 | [0.0006; 0.0176] | Positive |
| Romani, 2025 | 0.1961 | [0.0982; 0.3312] | Positive |
| Huang, 2024 | 0.2339 | [0.1794; 0.2959] | Positive |
| Garg, 2013 | 0.0 | [0.0000; 0.1684] | Positive |
| Perez, 2016 | 0.1887 | [0.1192; 0.2762] | Positive |
| Kuemmel, 2021 | 0.1068 | [0.0787; 0.1407] | Positive |
| Cheng, 2023 | 0.2917 | [0.1262; 0.5109] | Positive |
| Modi, 2019 | 0.0163 | [0.0034; 0.0469] | Positive |
| Saura, 2020 | 0.1056 | [0.0734; 0.1458] | Positive |
| Murthy, 2020 | 0.005 | [0.0006; 0.0178] | Positive |
| André, 2023 | 0.0593 | [0.0375; 0.0884] | Positive |
| Cortés, 2024 | 0.0428 | [0.0216; 0.0753] | Positive |
| Harbeck, 2024 | 0.127 | [0.0992; 0.1592] | Positive |
| Verma, 2012 | 0.0249 | [0.0130; 0.0432] | Positive |
| Sousa, 2022 | 0.0183 | [0.0074; 0.0373] | Positive |
| Krop, 2021 | 0.0307 | [0.0205; 0.0441] | Positive |
| Gianni, 2011 | 0.0093 | [0.0002; 0.0510] | Positive |
| Schneeweiss, 2013 | 0.0588 | [0.0163; 0.1438] | Positive |
| Hurvitz, 2017 | 0.0 | [0.0000; 0.0164] | Positive |
| Miles, 2021 | 0.1643 | [0.1455; 0.1845] | Positive |
| Urruticoechea, 2017 | 0.0413 | [0.0190; 0.0769] | Positive |
| Shaughnessy, 2021 | 0.0625 | [0.0304; 0.1119] | Positive |
| Tan, 2020 | 0.0357 | [0.0165; 0.0667] | Positive |
| Xu, 2021 | 0.0746 | [0.0364; 0.1330] | Positive |
| Martin, 2017 | 0.005 | [0.0020; 0.0102] | Positive |
| Hu, 2025 | 0.0 | [0.0000; 0.0166] | Positive |
| Hurvitz, 2013 | 0.0435 | [0.0091; 0.1218] | Positive |
| Bardia, 2024 | 0.0806 | [0.0568; 0.1104] | Low |
| Michalaki, 2017 | 0.0 | [0.0000; 0.1277] | Positive |
| Greil, 2024 | 0.0 | [0.0000; 0.0451] | Positive |
| Gelmon, 2015 | 0.0031 | [0.0001; 0.0172] | Positive |
| Krop, 2014 | 0.0149 | [0.0055; 0.0321] | Positive |
| Perez, 2017 | 0.0083 | [0.0017; 0.0241] | Positive |

CI, confidence interval; HER2, human epidermal growth factor receptor 2.

Supplementary Table 6. Subgroup analysis by agent used

| Study | Proportion | 95% CI | Agent |
| --- | --- | --- | --- |
| Wildiers, 2022 | 0.1951 | [0.0882, 0.3487] | Pertuzumab |
| Rugo, 2022 | 0.0568 | [0.0321, 0.0920] | Margetuximab |
| Yang, 2024 | 0.0833 | [0.0367, 0.1576] | Pertuzumab |
| Portera, 2008 | 0.5455 | [0.2338, 0.8325] | Pertuzumab |
| Xu, 2020 | 0.0328 | [0.0090, 0.0818] | Pertuzumab |
| Yamashita, 2021 | 0.0000 | [0.0000, 0.0725] | Pertuzumab |
| Gavilá, 2019 | 0.0241 | [0.0029, 0.0843] | Pertuzumab |
| Levicki, 2023 | 0.0769 | [0.0095, 0.2513] | TDM1 |
| Azambuja, 2024 | 0.0509 | [0.0419, 0.0613] | Lapatinib |
| Azambuja, 2023 | 0.1113 | [0.0989, 0.1246] | Pertuzumab |
| Morris, 2013 | 0.0303 | [0.0099, 0.0693] | Lapatinib |
| Dang, 2022 | 0.2191 | [0.1794, 0.2631] | Pertuzumab |
| Gherghe, 2022 | 0.2273 | [0.0782, 0.4537] | Pertuzumab |
| Tian, 2024 | 0.0680 | [0.0278, 0.1350] | Pertuzumab |
| Wang, 2022 | 0.4091 | [0.2071, 0.6365] | Pyrotinib |
| Yu, 2023 | 0.1408 | [0.0697, 0.2438] | Pertuzumab |
| Voort, 2021 | 0.6018 | [0.5340, 0.6669] | Pertuzumab |
| Krop, 2015 | 0.0338 | [0.0111, 0.0771] | TDM1 |
| Minckwitz, 2019 | 0.0135 | [0.0065, 0.0247] | TDM1 |
| Lenihan, 2011 | 0.1178 | [0.0851, 0.1575] | Pertuzumab |
| Modi, 2022 | 0.1968 | [0.1575, 0.2409] | TDXd |
| Ferre, 2020 | 0.1333 | [0.0376, 0.3072] | Lapatinib |
| Yuan, 2021 | 0.0400 | [0.0010, 0.2035] | Neratinib |
| Khoury, 2021 | 0.6333 | [0.4386, 0.8007] | TDM1 + Pertuzumab |
| Perez, 2008 | 0.0266 | [0.0215, 0.0324] | Lapatinib |
| Swain, 2020 | 0.0049 | [0.0006, 0.0176] | Pertuzumab |
| Romani, 2025 | 0.1961 | [0.0982, 0.3312] | Zanidatamab |
| Huang, 2024 | 0.2339 | [0.1794, 0.2959] | Pertuzumab |
| Garg, 2013 | 0.0000 | [0.0000, 0.1684] | Pertuzumab |
| Perez, 2016 | 0.1887 | [0.1192, 0.2762] | Pertuzumab |
| Kuemmel, 2021 | 0.1068 | [0.0787, 0.1407] | Pertuzumab |
| Cheng, 2023 | 0.2917 | [0.1262, 0.5109] | Pertuzumab |
| Modi, 2019 | 0.0163 | [0.0034, 0.0469] | TDXd |
| Saura, 2020 | 0.1056 | [0.0734, 0.1458] | Neratinib |
| Murthy, 2020 | 0.0050 | [0.0006, 0.0178] | Tucatinib |
| André, 2023 | 0.0593 | [0.0375, 0.0884] | TDXd |
| Cortés, 2024 | 0.0428 | [0.0216, 0.0753] | TDXd |
| Harbeck, 2024 | 0.1270 | [0.0992, 0.1592] | TDXd |
| Verma, 2012 | 0.0249 | [0.0130, 0.0432] | TDM1 |
| Sousa, 2022 | 0.0183 | [0.0074, 0.0373] | TDM1 |
| Krop, 2021 | 0.0307 | [0.0205, 0.0441] | TDM1 |
| Gianni, 2011 | 0.0093 | [0.0002, 0.0510] | Pertuzumab |
| Schneeweiss, 2013 | 0.0588 | [0.0163, 0.1438] | Pertuzumab |
| Hurvitz, 2017 | 0.0000 | [0.0000, 0.0164] | TDM1 + Pertuzumab |
| Miles, 2021 | 0.1643 | [0.1455, 0.1845] | Pertuzumab |
| Urruticoechea, 2017 | 0.0877 | [0.0544, 0.1322] | Pertuzumab |
| Shaughnessy, 2021 | 0.0625 | [0.0304, 0.1119] | Pertuzumab |
| Tan, 2020 | 0.0357 | [0.0165, 0.0667] | Pertuzumab |
| Xu, 2021 | 0.0746 | [0.0364, 0.1330] | Pyrotinib |
| Martin, 2017 | 0.0050 | [0.0020, 0.0102] | Neratinib |
| Hu, 2025 | 0.0000 | [0.0000, 0.0166] | ARX788 |
| Hurvitz, 2013 | 0.0435 | [0.0091, 0.1218] | TDM1 |
| Bardia, 2024 | 0.0806 | [0.0568, 0.1104] | TDXd |
| Michalaki, 2017 | 0.0000 | [0.0000, 0.1277] | Pertuzumab |
| Greil, 2024 | 0.0000 | [0.0000, 0.0451] | Pertuzumab |
| Gelmon, 2015 | 0.0031 | [0.0001, 0.0172] | Lapatinib |
| Krop, 2014 | 0.0149 | [0.0055, 0.0321] | TDM1 |
| Perez, 2017 | 0.0083 | [0.0017, 0.0241] | TDM1 |

CI, confidence interval

Supplementary Table 7. Subgroup analysis by type of therapy

| Study | Proportion | 95% CI | Type of Therapy |
| --- | --- | --- | --- |
| Wildiers, 2022 | 0.1951 | [0.0882, 0.3487] | Combination |
| Rugo, 2022 | 0.0568 | [0.0321, 0.0920] | Combination |
| Yang, 2024 | 0.0833 | [0.0367, 0.1576] | Combination |
| Portera, 2008 | 0.5455 | [0.2338, 0.8325] | Combination |
| Xu, 2020 | 0.0328 | [0.0090, 0.0818] | Combination |
| Yamashita, 2021 | 0.0000 | [0.0000, 0.0725] | Combination |
| Gavilá, 2019 | 0.0241 | [0.0029, 0.0843] | Combination |
| Levicki, 2023 | 0.0769 | [0.0095, 0.2513] | Monotherapy |
| Azambuja, 2024 | 0.0509 | [0.0419, 0.0613] | Combination |
| Azambuja, 2023 | 0.1113 | [0.0989, 0.1246] | Combination |
| Morris, 2013 | 0.0303 | [0.0099, 0.0693] | Combination |
| Dang, 2022 | 0.2191 | [0.1794, 0.2631] | Combination |
| Gherghe, 2022 | 0.2273 | [0.0782, 0.4537] | Combination |
| Tian, 2024 | 0.0680 | [0.0278, 0.1350] | Combination |
| Wang, 2022 | 0.4091 | [0.2071, 0.6365] | Combination |
| Yu, 2023 | 0.1408 | [0.0697, 0.2438] | Combination |
| Voort, 2021 | 0.6018 | [0.5340, 0.6669] | Combination |
| Krop, 2015 | 0.0338 | [0.0111, 0.0771] | Monotherapy |
| Minckwitz, 2019 | 0.0135 | [0.0065, 0.0247] | Monotherapy |
| Lenihan, 2011 | 0.1178 | [0.0851, 0.1575] | Combination |
| Modi, 2022 | 0.1968 | [0.1575, 0.2409] | Monotherapy |
| Ferre, 2020 | 0.1333 | [0.0376, 0.3072] | Combination |
| Yuan, 2021 | 0.0400 | [0.0010, 0.2035] | Monotherapy |
| Khoury, 2021 | 0.6333 | [0.4386, 0.8007] | Combination |
| Perez, 2008 | 0.0266 | [0.0215, 0.0324] | Monotherapy |
| Swain, 2020 | 0.0049 | [0.0006, 0.0176] | Combination |
| Romani, 2025 | 0.1961 | [0.0982, 0.3312] | Combination |
| Huang, 2024 | 0.2339 | [0.1794, 0.2959] | Combination |
| Garg, 2013 | 0.0000 | [0.0000, 0.1684] | Combination |
| Perez, 2016 | 0.1887 | [0.1192, 0.2762] | Combination |
| Kuemmel, 2021 | 0.1068 | [0.0787, 0.1407] | Combination |
| Cheng, 2023 | 0.2917 | [0.1262, 0.5109] | Combination |
| Modi, 2019 | 0.0163 | [0.0034, 0.0469] | Monotherapy |
| Saura, 2020 | 0.1056 | [0.0734, 0.1458] | Combination |
| Murthy, 2020 | 0.0050 | [0.0006, 0.0178] | Combination |
| André, 2023 | 0.0593 | [0.0375, 0.0884] | Monotherapy |
| Cortés, 2024 | 0.0428 | [0.0216, 0.0753] | Monotherapy |
| Harbeck, 2024 | 0.1270 | [0.0992, 0.1592] | Monotherapy |
| Verma, 2012 | 0.0249 | [0.0130, 0.0432] | Monotherapy |
| Sousa, 2022 | 0.0183 | [0.0074, 0.0373] | Monotherapy |
| Krop, 2021 | 0.0307 | [0.0205, 0.0441] | Combination |
| Gianni, 2011 | 0.0093 | [0.0002, 0.0510] | Combination |
| Schneeweiss, 2013 | 0.0588 | [0.0163, 0.1438] | Combination |
| Hurvitz, 2017 | 0.0000 | [0.0000, 0.0164] | Combination |
| Miles, 2021 | 0.1643 | [0.1455, 0.1845] | Combination |
| Urruticoechea, 2017 | 0.0877 | [0.0544, 0.1322] | Combination |
| Shaughnessy, 2021 | 0.0625 | [0.0304, 0.1119] | Combination |
| Tan, 2020 | 0.0357 | [0.0165, 0.0667] | Combination |
| Xu, 2021 | 0.0746 | [0.0364, 0.1330] | Combination |
| Martin, 2017 | 0.0050 | [0.0020, 0.0102] | Monotherapy |
| Hu, 2025 | 0.0000 | [0.0000, 0.0166] | Combination |
| Hurvitz, 2013 | 0.0435 | [0.0091, 0.1218] | Monotherapy |
| Bardia, 2024 | 0.0806 | [0.0568, 0.1104] | Monotherapy |
| Michalaki, 2017 | 0.0000 | [0.0000, 0.1277] | Combination |
| Greil, 2024 | 0.0000 | [0.0000, 0.0451] | Combination |
| Gelmon, 2015 | 0.0031 | [0.0001, 0.0172] | Combination |
| Krop, 2014 | 0.0149 | [0.0055, 0.0321] | Monotherapy |
| Perez, 2017 | 0.0083 | [0.0017, 0.0241] | Monotherapy |

CI, confidence interval

Supplementary Table 8. Subgroup analysis by cardiac monitoring frequency

| Study | Proportion | 95% CI | Cardiac Monitoring Frequency |
| --- | --- | --- | --- |
| Hans Wildiers | 0.1951 | [0.0882; 0.3487] | ≤8–9 weeks |
| Hope S. Rugo | 0.0568 | [0.0321; 0.0920] | ≤8–9 weeks |
| Ji-Xin Yang | 0.0833 | [0.0367; 0.1576] | >9 weeks |
| Chia C. Portera | 0.5455 | [0.2338; 0.8325] | ≤8–9 weeks |
| Binghe Xu | 0.0328 | [0.0090; 0.0818] | >9 weeks |
| Toshinari Yamashita | 0.0000 | [0.0000; 0.0725] | >9 weeks |
| Joaquín Gavilá | 0.0241 | [0.0029; 0.0843] | >9 weeks |
| Rea Levicki | 0.0769 | [0.0095; 0.2513] | >9 weeks |
| E. de Azambuja | 0.0509 | [0.0419; 0.0613] | >9 weeks |
| E. de Azambuja | 0.1113 | [0.0989; 0.1246] | >9 weeks |
| Chau Dang | 0.2191 | [0.1794; 0.2631] | >9 weeks |
| Anna van der Voort | 0.6018 | [0.5340; 0.6669] | ≤8–9 weeks |
| Ian E. Krop | 0.0338 | [0.0111; 0.0771] | >9 weeks |
| G. von Minckwitz | 0.0135 | [0.0065; 0.0247] | >9 weeks |
| S. Modi | 0.1968 | [0.1575; 0.2409] | ≤8–9 weeks |
| Roberto A. Leon-Ferre | 0.1333 | [0.0376; 0.3072] | >9 weeks |
| Yuan Yuan | 0.0400 | [0.0010; 0.2035] | ≤8–9 weeks |
| Katia Khoury | 0.6333 | [0.4386; 0.8007] | >9 weeks |
| Edith A. Perez | 0.0266 | [0.0215; 0.0324] | >9 weeks |
| Sandra M Swain | 0.0049 | [0.0006; 0.0176] | ≤8–9 weeks |
| Liang Huang | 0.2339 | [0.1794; 0.2959] | ≤8–9 weeks |
| Sainan Cheng | 0.2917 | [0.1262; 0.5109] | ≤8–9 weeks |
| S. Modi | 0.0163 | [0.0034; 0.0469] | ≤8–9 weeks |
| Cristina Saura | 0.1056 | [0.0734; 0.1458] | >9 weeks |
| R.K. Murthy | 0.0050 | [0.0006; 0.0178] | >9 weeks |
| Fabrice André | 0.0593 | [0.0375; 0.0884] | ≤8–9 weeks |
| Javier Cortés | 0.0428 | [0.0216; 0.0753] | >9 weeks |
| Nadia Harbeck | 0.1270 | [0.0992; 0.1592] | >9 weeks |
| Sunil Verma | 0.0249 | [0.0130; 0.0432] | >9 weeks |
| Romualdo Barroso-Sousa | 0.0183 | [0.0074; 0.0373] | >9 weeks |
| Ian E. Krop | 0.0307 | [0.0205; 0.0441] | >9 weeks |
| A. Schneeweiss | 0.0588 | [0.0163; 0.1438] | >9 weeks |
| Sara A Hurvitz | 0.0000 | [0.0000; 0.0164] | ≤8–9 weeks |
| D. Miles | 0.1643 | [0.1455; 0.1845] | ≤8–9 weeks |
| Joyce O’Shaughnessy | 0.0625 | [0.0304; 0.1119] | >9 weeks |
| Antoinette R Tan | 0.0357 | [0.0165; 0.0667] | ≤8–9 weeks |
| Binghe Xu | 0.0746 | [0.0364; 0.1330] | >9 weeks |
| Xichun Hu | 0.0000 | [0.0000; 0.0166] | ≤8–9 weeks |
| Sara A Hurvitz | 0.0435 | [0.0091; 0.1218] | >9 weeks |
| A. Bardia | 0.0806 | [0.0568; 0.1104] | >9 weeks |
| Karen A. Gelmon | 0.0031 | [0.0001; 0.0172] | >9 weeks |
| Ian E Krop | 0.0149 | [0.0055; 0.0321] | >9 weeks |
| Edith A. Perez | 0.0083 | [0.0017; 0.0241] | ≤8–9 weeks |

CI, confidence interval

Supplementary Figures

Supplementary Figure 1. **Funnel plot and Egger’s regression plot assessing small-study effects.** The upper panel shows the funnel plot of logit-transformed proportions versus standard error, with the pooled estimate indicated by the vertical line. The lower panel presents the Egger’s regression plot of standardised effect size against inverse standard error. Visual inspection suggests asymmetry, which may indicate small-study effects.

Supplementary Figure 2. **Funnel plot and Egger’s regression plot assessing small-study effects for relative risk estimates.** The upper panel shows the funnel plot of risk ratios against standard error, with the pooled estimate indicated by the vertical line. The lower panel presents the Egger’s regression plot of standardised effect size versus inverse standard error. Visual inspection suggests slight asymmetry, which may indicate small-study effects.

Supplementary Figure 3. **Subgroup analysis of pooled cardiotoxicity incidence by age.** Forest plots show study-specific proportions and pooled estimates for patients aged ≥55 years and <55 years. Squares represent individual study estimates with sizes proportional to inverse variance weights, and horizontal lines indicate 95% confidence intervals. The diamond represents the pooled estimate using a random-effects model. The red line indicates the prediction interval. Heterogeneity was assessed using the I² statistic, and subgroup differences were evaluated using a chi-square test.

Supplementary Figure 4. **Subgroup analysis of pooled cardiotoxicity incidence by drug class.** Forest plots display study-specific proportions and pooled estimates for monoclonal antibodies, antibody–drug conjugates, tyrosine kinase inhibitors, and combination regimens.

Supplementary Figure 5. **Subgroup analysis of pooled cardiotoxicity incidence by anthracycline exposure.** Forest plots display study-specific proportions and pooled estimates for patients with and without prior anthracycline use. were evaluated using a chi-square test.

Supplementary Figure 6. **Subgroup analysis of pooled cardiotoxicity incidence by HER2 status.** Forest plots display study-specific proportions and pooled estimates for HER2-positive and HER2-low populations.

Supplementary Figure 7. **Subgroup analysis of pooled cardiotoxicity incidence by individual HER2-directed agents.** Forest plots display study-specific proportions and pooled estimates for individual agents, including monoclonal antibodies, antibody–drug conjugates, and tyrosine kinase inhibitors.

Supplementary Figure 8. **Subgroup analysis of pooled cardiotoxicity incidence by type of therapy.** Forest plots display study-specific proportions and pooled estimates for combination therapy and monotherapy.

Supplementary Figure 9. **Subgroup analysis of pooled cardiotoxicity incidence by cardiac monitoring frequency.** Forest plots display study-specific proportions and pooled estimates for studies with monitoring intervals of ≤8–9 weeks and >9 weeks.

Supplementary Figure 10. **Leave-one-out sensitivity analysis for pooled cardiotoxicity incidence.** The plot shows the pooled estimates obtained by sequentially omitting each study to assess the robustness of the overall result. The pooled estimates are presented using a random-effects model, with 95% confidence intervals and prediction interval shown.
